# Evidence of nitrite acting as a stable and robust inducer of non-cultivability in *Mycobacterium tuberculosis* with physiological relevance

**DOI:** 10.1038/s41598-019-45652-8

**Published:** 2019-06-25

**Authors:** Suwarna P. Gample, Sonia Agrawal, Dhiman Sarkar

**Affiliations:** 1 CSIR-National Chemical Laboratory, Organic Chemistry Division, Pune, 411008 Maharashtra India; 2 Academy of Scientific and Innovative Research (AcSIR), Ghaziabad, 201002 India

**Keywords:** Bacteriology, Tuberculosis

## Abstract

Tuberculosis (TB) is the ninth leading cause of death worldwide, ranking above human immunodeficiency virus. Latency is the major obstacle in the eradication of this disease. How the physiology of the pathogen changes in transition to the latent stage needs to be understood. The latent bacteria extracted from animal hosts exist in a nonculturable (NC) phase, whereas bacteria extracted from most *in vitro* models are culture-positive. In the present study, we observed that nitrite, up to a concentration of 5 mM, shows the growth of *Mycobacterium tuberculosis* (MTB) in liquid media, but this effect starts reversing at higher concentrations. At a concentration of 10 mM, nitrite induces rapid nonculturability of MTB at the aerobic stage. This noncultivable dormancy was confirmed by analyzing the characteristics of NC bacteria. Further differential gene expression analyses clearly supported the formation of a dormancy phenotype. This study will be helpful for the use of this bacillus as a dormancy model in future studies on TB latency.

## Introduction

Tuberculosis (TB) is one of the most ancient infectious diseases of mankind and is one of the top 10 causes of death worldwide, affecting a third of the global population^1^. *Mycobacterium tuberculosis* (MTB) is the causative agent of TB, which can persist within humans for long periods of time without causing any symptoms^2,3^. Latent bacteria in the nonculturable (NC) stage cannot form colonies on solid media, which is why current diagnostic techniques are unable to detect more than 40% of TB recurrence cases. In addition, recent evidence suggests an association between nonreplicating bacilli and disease manifestations^4,5^. Recently, some reports showed that the development of drug resistance is not only because of the inheritable genetic resistance mechanism, but rather it is governed by changes in the physiological state of MTB^6^. However, more studies are needed in order to understand the metabolic processes that are critical for the pathogen to shift into dormancy and survive under stress conditions.

The factors that probably limit bacterial growth in granulomas include oxygen and nutrient deprivation, exposure to acidic pH, and the presence of nitric oxide or carbon monoxide^7–9^. On the basis of these factors, different dormancy models have been proposed. Dormant bacteria that were isolated from most *in vitro* models, such as the Wayne model and multiple stress model, were nonreplicating but remained cultivable^10,11^. However, bacteria obtained from *in vivo* models of latency, such as the Cornell model and noncultivable model developed by Shleeva *et al*. (2004), remained noncultivable^12,13^. It was observed that persistent and viable but noncultivable (VBNC) stages of MTB coexist and remain as part of the dormancy continuum. They could survive together as mixed population but differs in their physiological positions in the dormancy range^4^. VBNC cells have a higher toxin-to-antitoxin ratio as compared to persisters, so the VBNC stage could be considered a deeper stage of dormancy than persisters^4,14^. In all of these models, the external environment of the bacilli is modified. However, the internal targets whose modification plays a significant role in the development of noncultivability are still not fully explored.

However, in this study, we demonstrated that exposure of the MTB bacilli to nitrite induces dormancy under *in vitro* aerobic conditions. This dormancy is characteristically similar to the noncultivable type of dormancy, which was earlier reported and observed in isolates from *in vivo* animal models and humans. Thus, our study provides a new insight into the development of noncultivable dormancy in MTB.

## Results

### Effect of nitrite on the growth of MTB

**Figure 1:**
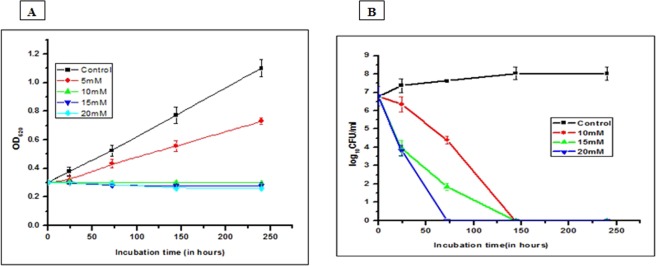
Growth kinetics of MTB in the presence of nitrite. The effect of nitrite on the growth of cells was determined by measuring (**A**) the OD at 620 nm and (**B**) the CFU count, at different time points with different nitrite concentrations. Log-phase cells treated with vehicle as a control (▪) and 5 mM (⚫), 10 mM (▴) 15 mM (▾), and 20 mM (♦) nitrite, respectively. More details are provided in the Materials and Methods section. The results are shown as the mean of three identical experiments ± standard deviation (SD). This experiment was repeated three times. The error bar represents the SD.

Sodium nitrite has been reported to inhibit the growth of a wide variety of bacteria, such as *Clostridia* and *Staphylococcus aureus*^15^. Thus, in order to assess the effect of nitrite on MTB, we measured the OD_620_ and colony forming unit (CFU) count of MTB bacilli at different time points. This study was carried out on MTB strain H37Ra, which has a >99.9% genome-wide similarity, and it was expected that the results will be applicable to pathogenic cells too. The results clearly indicated that nitrite supports the growth of MTB at concentrations below 5 mM, whereas at higher concentrations, nitrite significantly inhibits cell growth (Fig. 1A).

Further, a simultaneous assessment of the CFU count was performed at nitrite concentrations of 10, 15, and 20 mM at different time points. The CFU on an agar medium was also found to decrease by 3.23 log_10_ CFU/mL within 72 h of 10 mM nitrite treatment, with no CFU on day 6 (Fig. 1B). As compared to 7.37 ± 0.38 log_10_ CFU/mL on day 1, the CFU count of the control was found to be 8.03 ± 0.33 log_10_ CFU/mL on day 6. The reduction in the CFU count raises the possibility of death or acquired nonreplicative feature of the bacilli.

### Effect of nitrite on the viability of MTB bacilli

**Figure 2:**
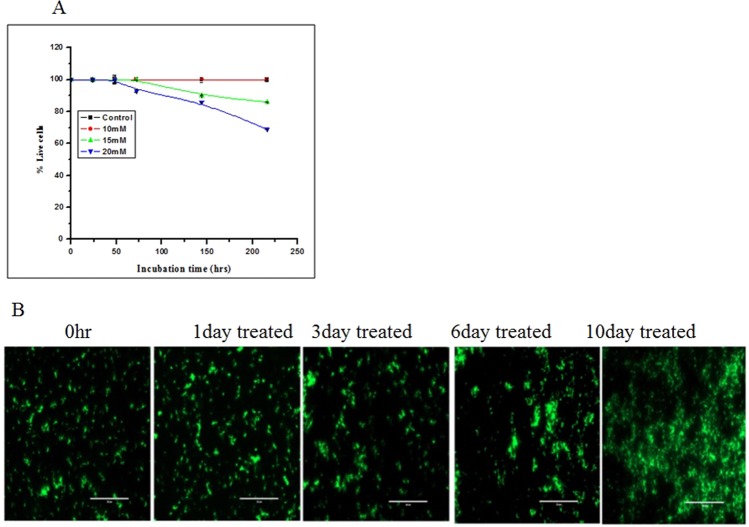
Relative viability analysis of MTB cells. (**A**) Bacterial cells were exposed to 10 mM (⚫), 15 mM (▴), and 20 mM (▾) nitrite, and percent viability was analyzed up to 10 days of treatment with respect to the untreated control (▪) as 100% viable. The experiment was performed in triplicate. Data are shown as the mean ± SD. (**B**) Fluorescence microscopic images of cells treated with nitrite (10 mM) were captured at different time intervals using an EVOS microscope as mentioned in the Materials and Methods section. The data shown is representative of three independent experiments (scale bar: 50 µm).

In order to further elucidate the reason behind the observed decrease in the CFU, we performed live–dead staining of these bacilli. Bacilli treated with nitrite (10 mM) remained 100% viable for more than 10 days without forming any colonies on agar (Figs 1B and 2A,B). However, treatment with higher concentrations of nitrite also did not reduce the cell viability of MTB cells in any significant manner.

### Effect of nitrite on the morphological properties of MTB

**Figure 3:**
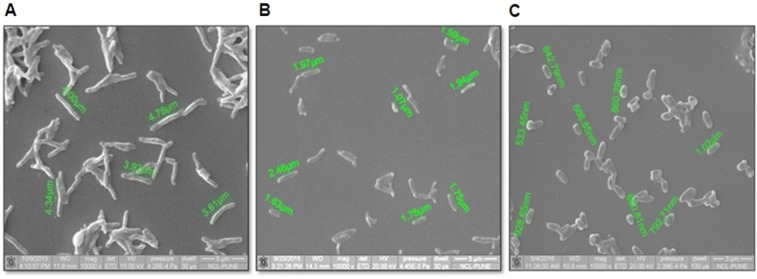
Electron microscopic measurement of nitrite-treated and nontreated MTB cells. (**A**) The log-phase culture was treated with nitrite (10 mM), and SEM images were taken on (**B**) day 3 and (**C**) day 6 of treatment. Scale bar: 1 µm. More details on the experiment were described in the Materials and Methods section.

It is well known that the shape of the bacilli changes to more rounded with a concomitant reduction in their size when actively growing cells shift to the NC state^11,16,17^. The morphological characteristics of noncultivable bacilli developed after treatment with nitrite were analyzed using scanning electron microscopy (SEM). It was observed that nitrite-treated cells decreased in size compared to untreated control cells. Interestingly, on day 6, the size of the cells treated with nitrite (10 mM) was reduced to a level between 0.8 and 1.2 µm, as compared to actively growing controls (size: 3.0–6.0 µm) (Fig. 3, Table 1).

**Table 1:** Distribution of the bacilli sizes in the presence of nitrite.

Length (µm)	Cell percentage		
	Control	Nitrite (day 3)	Nitrite (day 6)
1 ± 0.5	2	7	98
2 ± 0.5	6	64	2
3 ± 0.5	29	29	0
4 ± 0.5	41	0	0
5 ± 0.5	13	0	0
6 ± 0.5	9	0	0

After analyzing the cell size distribution (Table 1), it was found that the size of 98% of the cells was between 0.6 and 1.5 µm on day 6 of nitrite treatment. However, in the controls, the size of 41% of the cells was in the range of 3.5–4.5 µm, and only 2% of the cells were in the range of 0.6–1.5 µm. This observation strengthened the notion of the reduction of cell size in nitrite-treated MTB cells being a characteristic feature of the VBNC stage.

### Loss of acid fastness in nonculturable cells

**Figure 4:**
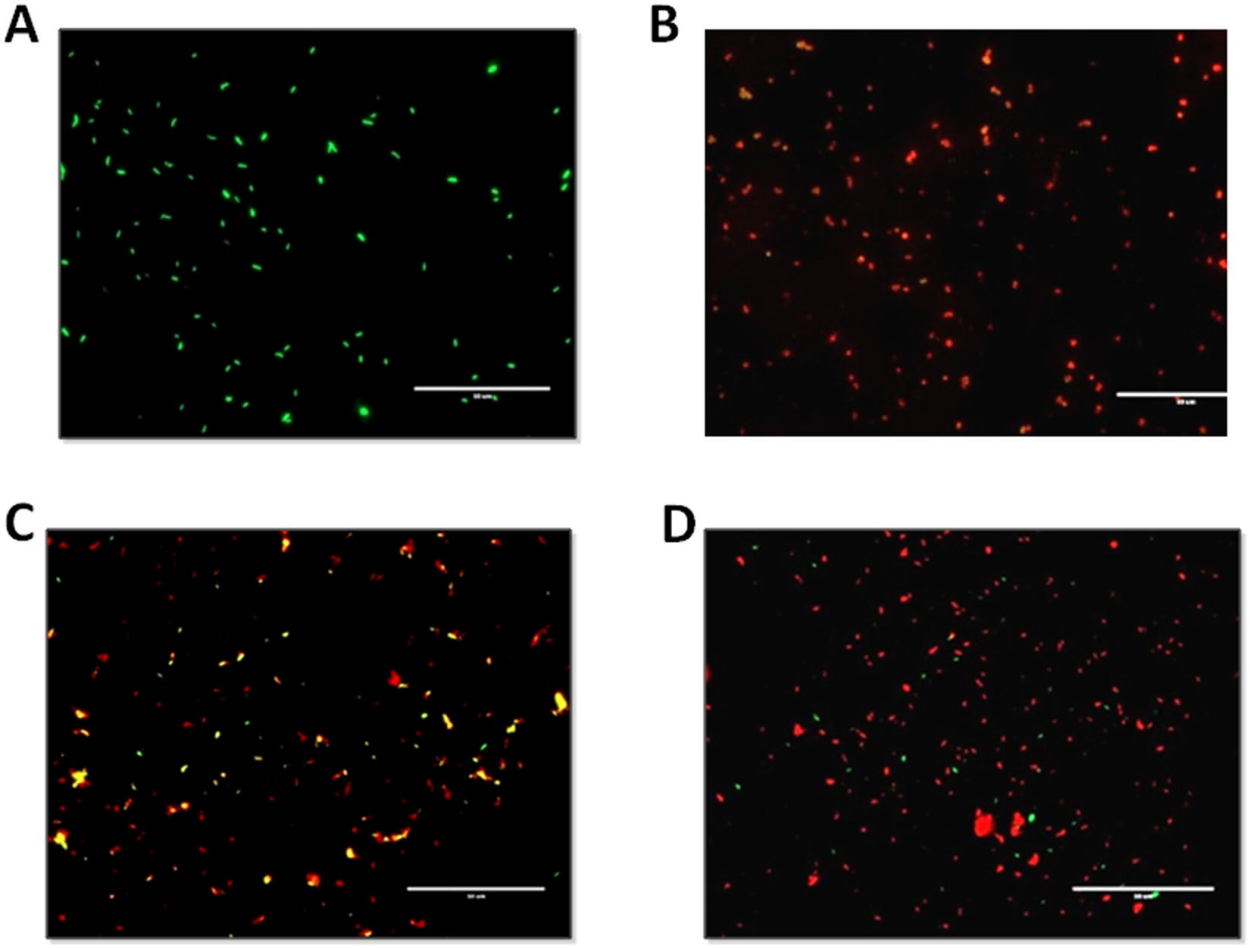
Effect of nitrite exposure on the acid-fast property of MTB bacilli. MTB cells were stained with Auramine O and Nile Red fluorescence dyes at different time intervals. (**A**) Untreated cells were used as the vehicle control. (**B**) NRP stage 2 culture of the Wayne model was stained as a positive control. Cells treated with nitrite (10 mM) were stained and observed on day 3 (**C**) and day 6 (**D**) of treatment. More details are described in the Materials and Methods section. Fluorescence microscopic images were captured at 60× objective using an EVOS microscope (Life Technologies) (scale bar: one unit = 50 µm).

Loss of acid fastness is another major characteristic change that takes place when mycobacteria shift from the active state to a dormant state. Thus, in order to support our observation of shifting of bacilli to the noncultivable dormant state upon nitrite treatment, we assessed the acid-fast property using the fluorescent acid-fast staining dye Auramine O in combination with the neutral lipid staining Nile Red dye. The log-phase culture and NRP stage 2 culture of the Wayne model were used as two extreme controls (Fig. 4A,B).

However, with respect to the controls (Fig. 4A), cells treated with nitrite (10 mM) showed red fluorescence after six days, indicative of the loss of the acid-fast property of the bacilli (Fig. 4D). Interestingly, cells stained after three days of treatment were observed to have yellow fluorescence due to the combined effect of Auramine O and Nile Red stains, indicative of the gradual shift of the acid-fast property from positive to negative (Fig. 4C).

### Effect of nitrite on the antibiotic sensitivity of MTB

**Figure 5:**
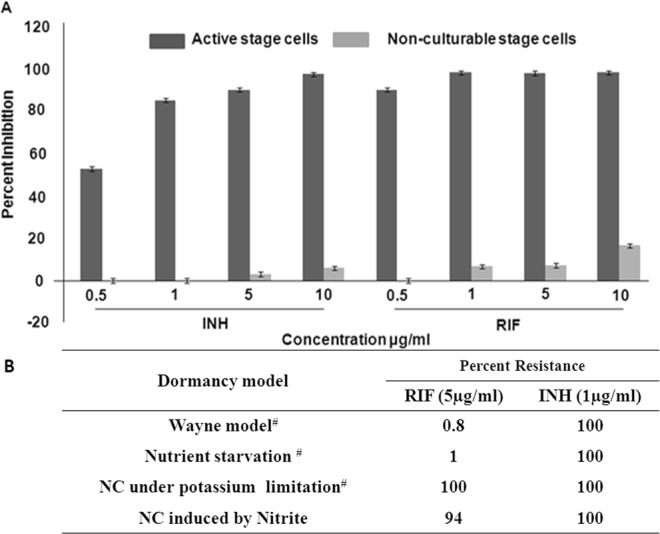
Determination of the sensitivity of nitrite-treated bacilli to antitubercular drugs. (**A**) Actively growing and nitrite-treated MTB cells were treated with RIF and INH at different concentrations (0.5, 1, 5, and 10 µg/mL) for seven days, and viable bacilli were enumerated by the live–dead staining method. (**B**) A table depicting a comparison between the percent viable cells that remained with other dormancy models after independent treatment with 5 µg/mL INH and RIF each. The details of the protocol followed in both experiments were described in the Materials and Methods section. ^#^Percent resistance values taken from *Antimicrob Agents Chemother*. 2014.

Resistance to antibiotics is a hallmark of latency in mycobacteria^7,18–20^. Standard antimycobacterial drugs like rifampin (RIF) and isoniazid (INH) were used to assess the sensitivity of nitrite-treated cells. Nitrite-treated MTB cells showed total resistance toward standard antitubercular drugs like RIF and INH, even at concentrations higher than IC_90_ (Fig. 5A). These results were in accordance with most of the dormancy models, including few that demonstrated significant susceptibility toward RIF (Fig. 5B). RIF inhibits RNA synthesis, which is essential for dormant cells at a low level^6^. On the other hand, INH impacts cell wall biosynthesis, a process that is inactive in dormant cells^15^. Thus, cells in all dormancy models were expected to be characterized by their resistance to INH.

### Transcriptional response of MTB bacilli to nitrite stress

The effect of nitrite on gene regulation of MTB was analyzed using microarray analysis. The gene expression pattern on days 1, 3, and 6 of nitrite treatment was compared with the transcriptome data obtained from log-phase cells without nitrite treatment as a control. On day 6, in nitrite-treated cells, 30 genes were upregulated and 110 genes were downregulated, as compared to untreated cells. However, on days 1 and 3 of nitrite treatment, 107 and 98 genes were upregulated, whereas 8 and 50 genes were downregulated, respectively. With the increase of the duration of nitrite treatment, the number of upregulated genes decreased, with a simultaneous increase in the number of downregulated genes (Tables S1–S6).

On day 1, as compared to the control, more genes were induced in nitrite-treated cells, possibly to cope up with the stress condition. However, on day 6, the number of downregulated genes was high, which might be due to the low metabolic state of bacteria.

### Overlap between differentially expressed genes of MTB

The number of overlapping genes between the three data points (1, 3, and 6 day nitrite treated) is depicted as a Venn diagram (Fig. S1). Among the differentially expressed genes, *ppe65*, *Rv1815*, and *mce1c* were repressed (Table S6), whereas the *rrl*, *rnpB*, and *cysK2* genes were induced (Fig. S1B), at all three time points after nitrite treatment. The *cysK2* gene is involved in the cysteine biosynthetic pathway. Sulfur metabolism and *de novo* cysteine biosynthesis are important for redox homeostasis in persistent MTB, and these pathways could provide promising targets for novel antibiotics for the treatment of the latent form of the disease^21^.

### Comparative profile of overlapping genes between nitrite Treatment and other dormancy models

The differentially regulated genes identified after nitrite treatment were compared with other dormancy models. The gene expression profile in nitrite-treated cells exhibited a significant difference between overlapping genes with other dormancy models (Table 2). From the analysis, it was observed that the expression pattern obtained from most of the dormancy models as well as on days 1 and 3 of nitrite treatment has indicated similarity from the higher number of overlapping genes compared to day 6. The NC cells obtained in a potassium deficiency model and on day 6 of nitrite treatment showed a high number of overlapping genes, as compared to days 1 and 3 of nitrite treatment.

**Table 2:** Comparative profile of overlapping genes between nitrite treatment and other models.

Condition	Genes regulated	Percentage of overlapping genes		
		Day 1	Day 3	Day 6
Iron depletion^29^	93	35	12	10
Hypoxia (0.2% O_2_ for 2 h, H37Rv)^30^	398	5	3	7
Nitric oxide (50 µM DETA/NO)^65^	48	0	10	12
Nitrate respiration (5 mM)^28^	113	11	14	16
Log-phase −*K* compared to log-phase +*K*^10^	182	4	3	2
Stationary phase −*K*+ compared to stationary phase *K*+^10^	51	2	4	10
Macrophage phagosome 2 h after infection^22^	68	9	3	0
From 5 min to 2 h after infection^22^	118	7	2	0
Persistence within macrophage^22^	115	11	3	7
Vitamin-C-triggered dormancy (10 mM)^66^	563	7	3	5

The total number of genes has shown the similar pattern of expression in nitrite-treated and other models were denoted. A representation factor >1 was selected and represented in the table. This representation factor shows that the overlap that occurred is real and not by chance.

### Tracing of pathways involving Up- and downregulated genes

The differentially expressed genes were classified into various functional categories, such as lipid metabolism, transcription, translation, and carbohydrate metabolism. Some of the functional classes that showed changes after nitrite treatment are discussed below.

#### Chaperones and heat shock proteins

Genes under the *devR* regulon are upregulated under stress conditions such as hypoxia, oxidative stress, pH stress, and bacteria infecting macrophages^22–26^. In the presence of nitrite, the *dosR* regulon gene *hsp* (heat shock protein) was upregulated on days 1 and 6, whereas the *hspX* gene was downregulated on days 3 and 6. The *hsp* gene gets induced generally under all stress conditions and 24 h after infection of macrophages^23^.

The *HspX* protein showed low abundance in potassium-deficient NC cells, also downregulated under acidic conditions^27^. 21 *dosR*-regulon-related genes in potassium-deficient cells and 27 genes in nitrate-treated cells were downregulated^10,28^. *The expression dosR* regulon is not found to change in response to H_2_O_2_, in the persisting cells during starvation, as well as the gradual depletion of the carbon source^16,24^. In recent literature, it has been hypothesized that the *dosR* regulon is induced when oxygen tension is reduced in aerobically respiring bacteria. In this study, the cells did not experience oxygen stress, which could be preventing the induction of *dosR* regulon as observed in other dormancy models.

#### Detoxification-related genes

*ahpC* is an alkyl hydroperoxide reductase, which is important for resistance against oxidative stress and is induced in different dormancy models^23,24,29–32^. The *ahpC* gene was found to be upregulated in MTB bacilli on day 1 of exposure to nitrite.

Sulfur metabolism genes like *cysA2*, *cysK1*, and *cysH* were found to be upregulated on day 1, whereas the *cysK2* gene was found to be upregulated at all three time points. The sulfur assimilation genes *cysD* and *cysN* are induced during iron deficiency, in oxidative stress, and in the presence of a vitamin-C-induced dormancy condition^33^, whereas the *cysH* gene is also induced in the iron deficiency condition^29^. Bacteria regulate sulfur assimilation in response to toxic oxidants, and the cell protects itself from reactive species by the induction of these genes. It was reported that sulfur metabolism is involved in the antioxidant defense mechanism of MTB^34^.

#### Lipid and mycolic acid metabolism

Genes related to the fatty acid degradation pathway, such as *fadD9* and *fadD31*, coding for fatty acid CoA ligase, and genes *adhD*, *alkB*, and *adhC* were upregulated on day 1, whereas genes *fadA2* and *Rv0914C* were downregulated on day 3. Like other dormancy models, mycobacteria alter their own metabolism to use fatty acid as a carbon source after nitrite treatment^10^.

Genes involving fatty acid biosynthesis were downregulated on days 3 and 6. As fatty-acid-synthesis-related genes were downregulated from day 3 onwards, the bacilli may have reduced their energy consumption required for fatty acid synthesis. The *acpM* gene, which is involved in mycolic acid biosynthesis, and the *wag31* gene (cell wall synthesis protein) were also downregulated on day 6^35,36^. *wag31* regulates the cell morphology of bacteria and is involved in the oxidative stress response of MTB^36^. The *ald* gene, which catalyzes alanine hydrolysis, an important constituent of the peptidoglycan layer, is upregulated on days 1 and 6, which can be correlated with the morphological changes that occurred in MTB after nitrite treatment.

Many dehydrogenases genes like *ald* (alanine dehydrogenase), *icd2* (isocitrate dehydrogenase), *fadB2* (3-hydroxybutyryl-CoA dehydrogenase), *adhD* (alcohol dehydrogenase), *pdhB* (putative 2-oxoisovalerate dehydrogenase), and *lldD2* (lactate dehydrogenase) were downregulated on day 6. This might be needed to maintain the NADH pool under dormant conditions.

#### *Replication*, *transcription*, *and translation*

In consensus with the earlier reports on NC cells^10^, a large number of genes involved in replication, transcription, and translation were downregulated in the presence of nitrite^37,38^. In this study, the DNA replication initiation protein *dnaA* was downregulated on day 6. A differential expression of transcriptional factors was observed in nitrite-treated cells. The genes *sigB*, *sigH*, and *sigE* were upregulated on day 1, whereas *Rv0516c* was downregulated on day 3 and anti-sigma E factor RseA encoding *Rv1222* was upregulated on day 6. *sigH* may be involved in the response of MTB toward reactive oxygen species (ROS) and reactive nitrogen intermediates (RNIs). During the transition to the NC state, some marker genes of stress conditions were induced, such as *hsp*, the chaperones *Rv0440* and *Rv3417с*, and sigma-factors like *sigG* and *sigE*. *sigG* regulates the genes necessary for survival inside macrophages, whereas *sigB* controls the stationary phase regulon and general resistance to stress^16^.

*recC* (exodeoxyribonuclease V) and *Rv3828c* genes involved in recombination were upregulated on day 3, whereas *Rv3750C* was downregulated on day 6. The DNA repair mechanism may become activated after nitrite treatment to protect the DNA under stress conditions.

Translation-related genes like IF-3, IF-1, *tuf*, and *fusA1* were downregulated on day 6. 16 proteins of 30 S and nine proteins of 50 S ribosomal encoding genes were downregulated on days 6 and 3, respectively. Overall, DNA replication, transcription, ribosome assembly, translation, and purine and pyrimidine metabolism were downregulated in nitrite-treated MTB bacilli.

#### *Gluconeogenesis*, *tricarboxylic acid (TCA) cycle*, *and glyoxylate metabolism*

In the persistent phase, metabolism is shifted from glucose to lipids, glycolysis is downregulated, and the glyoxylate shunt is upregulated, allowing anaplerotic maintenance of TCA cycle^39–41^.

In the presence of nitrite, gluconeogenesis and TCA cycle genes were upregulated on day 1. The genes involved in glyoxylate metabolism and pyruvate metabolism were also upregulated on day 1, and then downregulation was observed. It can be concluded that, from the first day of nitrite treatment, the cells shifted from glucose to lipids as a source of carbohydrate.

The *ndh* gene encoding NADH dehydrogenase and *nirA* protein involved in sulfate reduction were upregulated on day 1. During aerobic and nitrate respiration, NADH dehydrogenase I (NDH-2) enzyme is used^42^. It was reported that MTB also utilizes substrates like nitrate for respiration at the dormant state^33,43–45^. It has been shown that, during the NC stage, bacteria respire anaerobically, even though oxygen is available, with uncoupled non-proton-pumping NADH dehydrogenase II playing an important role.

#### Regulators

Transcriptional regulators like *Rv0485*, *Rv2621C*, and *Rv3249c* (TetR family transcriptional regulators); *Rv2499c* (oxidase regulator); and *whiB3* (redox-responsive transcriptional regulator) were upregulated on day 1. *hrcA* (heat-inducible transcriptional repressor) and *ESX-1* (transcriptional regulator *EspR*) were upregulated on day 3 and the ArsR family transcriptional regulator was upregulated on day 6, whereas HTH and *Rv2166* gene encoding the transcriptional regulator MraZ were downregulated on day 6.

The *furA* gene product negatively regulates the iron scavenging response, is induced under oxidative stress and hypoxia conditions, and was upregulated on day 6^10,23,26^. The same gene was upregulated in the presence of vitamin C, which suggested that an iron-deficient environment may aid in the repair/replacement of iron-containing proteins that may be damaged during stresses caused by ROIs and RNIs^33^.

The *rpfC* gene encoding resuscitation promoting factor was downregulated on days 3 and 6. Other dormancy models even show repression of the resuscitation promoting factor^24^.

#### ESAT-6 secretion system

The ESAT-6 secretion system 1 (ESX-1) is associated with the virulence and pathogenesis of MTB^46,47^. 10 ESAT-protein-encoding genes were upregulated on day 1, whereas three and seven genes were downregulated on days 3 and 6, respectively. It was reported that, at a dormant state, ESAT protein abundance is low, and a similar result was found in cells treated with nitrite for six days^27,29,47^.

#### PE and PPE family protein

A variable gene expression pattern was observed in nitrite-treated cells. Most of the genes were upregulated on day 1, whereas similar genes were downregulated on days 3 and 6. These proteins show a variable expression in MTB-infected macrophages and the mouse model, which could provide a dynamic antigenic profile during changing microenvironments within the host^48–50^.

#### Transmembrane proteins

Six genes encoding integral or transmembrane proteins were upregulated on day 1, and the *ftsK* gene a transmembrane protein encoding for the cell division was upregulated on day 3. Similar types of transmembrane proteins were also found to be upregulated under low-iron conditions, 24 h after infection to the macrophage, hypoxia at 2% O_2_ for 2 h^20,23,29^. Similarly, four membrane-protein-coding genes were upregulated on day 6. Novel pathways involving membrane proteins play an important role in the transition of growing cells to the NC stage^10^. After nitrite treatment, most of the transmembrane proteins are upregulated and may be involved in the transition of bacilli from the active to the NC state.

#### Toxin–antitoxin system

The gene *rv2063* encoding antitoxin MazE7 was upregulated on day 3. Antitoxin VapB14 encoding genes *Rv0968* and *Rv1955* coding for toxin HigB were upregulated on day 6, and *Rv3408* gene coding for ribonuclease VapC47, a toxin, was downregulated on day 6. The available literature shows that the toxin–antitoxin system plays a role in the stress physiology of bacteria, and it could be part of the specific mechanism involving different enzymes and regulators. Activation of this system results in the shutdown of protein synthesis, until favorable conditions occur, and this system is also involved in the transition of bacteria from the active to a dormant and NC phenotype^14,51–55^.

Based on the results of the transcription analysis of the noncultivable cells obtained in our study (treated with nitrite for six days) and those obtained in other models of persisters, some genes were found to be common, which are listed in Table 3. These genes may play an important role in the noncultivable state of MTB.

**Table 3:** Common genes found in different models.

ORF	Genes	NRP stage 2 Wayne^16^	Persistence within macrophages^16^	Artificial granuloma in mice^16^	NC state K^+^ limited stationary phase^16^	Nitrite treatment (day 6)
Rv0188		0.8	2.8	2.7	2.5	1.93
Rv0251C	*hsp*	4.5	5.6	3.9	4.5	2.97
Rv0191		2	1.8	—	2.8	1.52
Rv2497C		3.4	2.1	2	4	2
Rv2710	*sigB*	—	3.8	4.7	4.6	2.94
Rv2780	*ald*	6.1	2.4	2.4	4.9	1.77
Rv3290C	*lat*	3.6	7.5	5.6	4	2.84

#### Validation of microarray data by quantitative polymerase chain reaction (qPCR)

**Figure 6:**
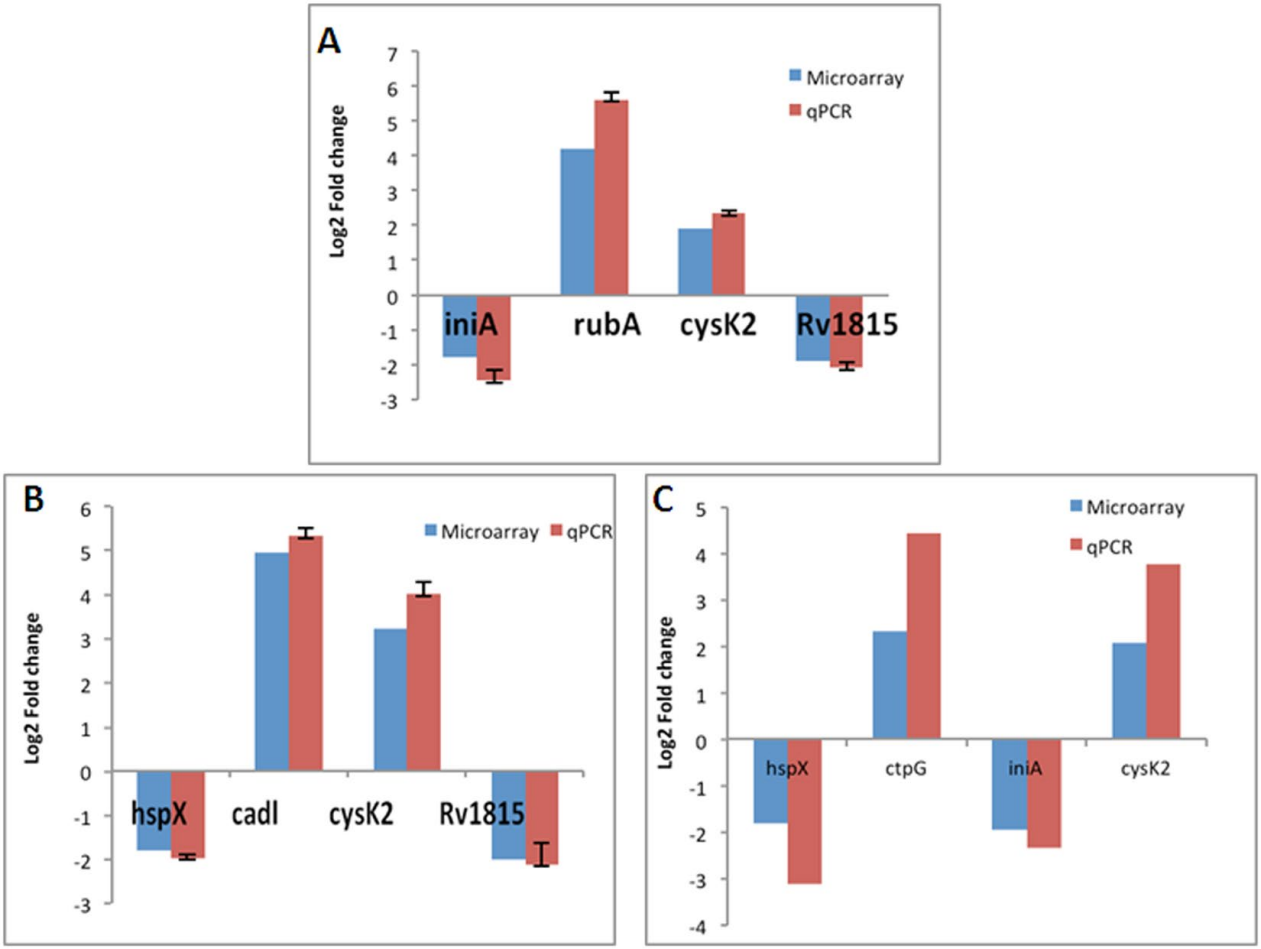
Differential gene expression level measurement by microarray and qPCR. The gene expression levels of nitrite-treated cells after (**A**) one, (**B**) three, and (**C**) six days were measured using microarray and qPCR. The expression level of the genes was compared with aerobically grown log-phase cells (control). The blue-colored column shows the fold change obtained by microarray, whereas the red-colored column shows the fold change obtained by qPCR for each respective gene. Each experiment was performed three times, and the SD is shown on the graph.

In order to validate the microarray data, the fold change of selected genes was determined using qPCR. Even though the fold change measured by the qPCR was higher than that measured by microarray, a similar pattern of gene expression was observed by both methods (Fig. 6).

## Discussion

It is well known that bacilli can survive the latency stage within the host for many years. Therefore, it can be assumed that the endogenous inducer should be chemically and metabolically very stable. Among the known factors like oxygen depletion, nutrient starvation, acidic pH, CO, and NO, cannot be sourced for years within the host milieu to maintain the non-replicating status of the bacilli. Nitrate and nitrite are the most predominantly available RNIs observed in the lung cavities. However, the possibility of nitrite or nitrate playing a major role is not yet understood. Nitrite has been known to interfere with energy conservation by inhibiting oxygen uptake, oxidative phosphorylation, and proton-dependent active transport, where it acts as an uncoupler, causing the collapse of the proton gradient. Nitrite has also been known to inhibit intracellular thiol groups and certain metabolic enzymes like glycolytic enzymes^2^. Endogenously generated nitrite may be beneficial for host cells to survive in hostile environments^28,56^.

In the present study, nitrite was added to an MTB culture under aerobic conditions. As reported in the literature, the effect of nitrite was observed on MTB strain H37Ra^28^. The concentration of nitrite and strain used in the study was different in our experiments than reported earlier. We observed that the bacteriostatic effect of nitrite appears on MTB with the sharp reduction in the CFU count, without any adverse effect on the viability of cells (Figs 1A,B and 2A,B). Moreover, when these nitrite-treated bacilli were inoculated into nitrite-free media, there was no growth on both the Dubos broth and agar plate. Thus, it can be concluded that nitrite restricts the growth of MTB bacilli, probably shifting it to a noncultivable state. Owing to the similarity of nitrite-treated MTB cells to the bacilli isolated from patients with TB^57^ and infected animals, a comprehensive study of these forms will be important to elucidate the molecular mechanisms that underlie the development and maintenance of nonreplicating persistence of mycobacteria.

Furthermore, development of antibiotic resistance is associated with the loss of acid fastness. For example, INH treatment impacts cell wall biosynthesis, a process that is inactive in dormant cells^58^. As a result, cells in all dormancy models are expected to be characterized by their resistance to INH. Moreover, most of the dormancy models demonstrated significant susceptibility to RIF. In our study, under nitrite stress, nearly all of the MTB cells with lipid droplets failed to show acid-fast staining and developed phenotypic antibiotic resistance during the development of dormancy (Figs 4 and 5).

**Figure 7:**
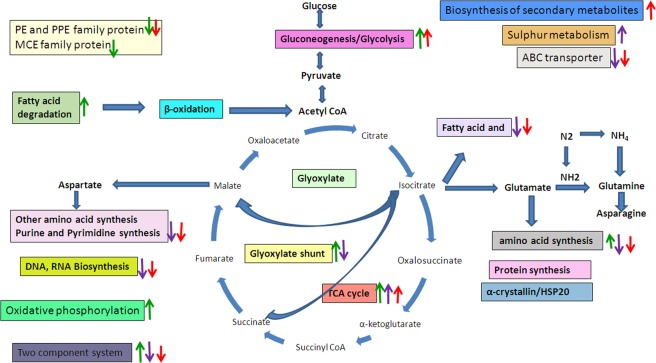
Effect of nitrite on the metabolic pathways of MTB. Nitrite affects the metabolic pathways of MTB. Nitrite treatment given for one (), three (), and six () days alters the metabolic pathways, which is shown in the figure. The figure shows the upregulation and downregulation of genes involved in the marked pathway.

Global changes in the gene expression level occurred in nitrite-induced dormant MTB. As reported earlier, we found a similar shift in metabolism from the available set of genes and proteins when compared between the active and dormant states^6,10,28,33^ (Table 2, Fig. 7).

From the first day of nitrite treatment, genes involved in fatty acid degradation were upregulated and fatty-acid-synthesis-related genes were downregulated. This indicates that cells shifted from glucose to lipids for an energy source and confirms the previous evidence^59^. Genes encoding proteins such as the ABC transporter and ATP binding protein were downregulated on day 6. Four genes encoding dehydrogenases were upregulated, which are required to maintain the NADH pool under dormant conditions^60^.

The 34 genes involved in DNA replication, transcription, ribosome assembly, translation, and purine and pyrimidine metabolism were downregulated after day 3 of nitrite exposure. Thus, it is very clear that the cell was investing minimum energy for cell division/replication^33^.

During the transition to the NC state, some genes like *hsp*, *Rv0440*, *Rv3417с*, *sigG*, and *sigE*, used as markers of stress conditions, were induced in the presence of nitrite. *dosR*-regulon gets induced under dormant conditions, but recently it was reported that it acts as a regulator of oxygen tension and gets induced under hypoxia conditions^16,30^.

The *wag31* gene, member of the *nrdHIEF* operon genes, involved in the oxidative stress response of MTB, also protects cells from ROS and regulates the cell morphology of bacteria. It was observed that transmembrane proteins were upregulated after nitrite treatment, which may be involved in the change in morphology of bacteria during the transition from the active to the dormant state^33,61^.

Transcriptional regulators (nine genes) and members of a two-component system (six genes) were differentially expressed in response to nitrite exposure. These proteins are involved in many cellular functions, such as the control of multidrug efflux pumps; pathways for the biosynthesis of glyoxylate shunt; and responses to osmotic stress, environmental stimuli, oxidative stress, and toxic chemicals, and are involved in the transition of cells from the active to the dormant state. Thus, these factors could lead us to understand the signaling processes that are necessary for survival under stress conditions.

Redox-sensitive proteins, namely, ferredoxin, thiosulfate sulfur transferase, and methyl transferase, which maintain cellular redox homeostasis, were found to be downregulated^62^. All of these results showed that the bacilli possess a well-equipped machinery to survive under stress conditions. From the transcriptional study of our model and other models, some common genes were pinpointed in Table 3, which may play an important role in the survival of NC bacteria. These genes can be further studied as good drug targets against latent MTB.

In summary, exposure of cells to nitrite-induced noncultivable dormancy. Moreover, noncultivability was well achieved in our nitrite-induced dormant culture. The rapid noncultivability and dormancy phenotype in nitrite-treated MTB bacilli could characteristically make it one of the most favored dormancy models because of its very similar characteristics to those of cells obtained from clinical samples. This finding could be useful in designing new *in vitro* and *in vivo* models for studying latency and screening of potent and specific therapeutic agents against TB. The major advantage of using a nitrite-induced dormant culture is that a large amount of noncultivable cells can be obtained within a short period of time using this robust protocol. In addition, this report mainly helps understand the metabolic shift that occurs during the dormancy of tubercular bacilli. Thus, the presence of nitrite in the medium could act as an extracellular robust inducer of noncultivable dormancy in MTB.

## Materials and Methods

### Organism and media used

MTB strain H37Ra was a gift from AstraZeneca, Karnataka, India. All chemicals were purchased from Sigma (USA), unless otherwise mentioned. MTB was cultured in a *Mycobacterium phlei* medium containing 0.5 g of potassium dihydrogen orthophosphate, 0.25 g of sodium citrate, 60 mg of magnesium chloride, 0.5 g of asparagine, and 2% (v/v) glycerol in 100 mL of distilled water at pH 6.6 ± 0.2. The stock culture was maintained at −70 °C and subcultured once in a liquid medium before inoculation into an experimental culture.

### Cultivation of aerobic bacilli

For aerobic cultivation, bacterial cultures were grown in a 30 mL defined medium in a 100 mL flask under aerobic conditions in a shaker incubator (Model 481; Thermo Electron Corporation, Waltham, MA, USA) maintained at 150 rpm and 37 °C until the logarithmic phase (OD_620_ ~ 1.0) was reached.

### Growth kinetics of MTB in the presence of nitrite

Different concentrations (5, 10, 15, and 20 mM) of nitrite were added when the cell OD_620_ reached 0.3, and its effect on growth was monitored by reading the absorbance of the culture at 620 nm as well as by counting the CFU.

### Estimation of the cell viability assay

Viability was measured using a live–dead (*Bac*Light) staining kit (Molecular Probes, Eugene, OR, USA) following the manufacturer’s instructions. The assay utilizes a mixture of SYTO 9 green fluorescent nucleic acid stain and red fluorescent nucleic acid stain, propidium iodide (PI). SYTO 9 stains both live and dead cells having an intact or damaged membrane, whereas PI stains dead cells or dying cells. Briefly, log-phase cells were grown in a *M*. *phlei* medium up to an optical density of 0.2 (620 nm) and treated with nitrite at different concentrations (10, 15, and 20 mM). The samples (200 µL) were incubated with SYTO 9 (5.1 μM) and PI (80 μM) for 15 min. Fluorescence was scanned, keeping excitation fixed at 488 nm, with emission spanning from 490 to 700 nm. In addition to this, cells treated with nitrite (10 mM) were further used for imaging analysis. The cells were first washed with phosphate-buffered saline and suspended in a fresh medium. A smear was then prepared with 10 µL of cell suspension on a grease-free slide, and a fluorescence image was taken using an EVOS microscope (Life Technologies, Darmstadt, Germany). Standardization of both the dyes and the exposure time was performed with respect to 100% live and 100% dead MTB cells. (Fig. S2). Representative data for INH killing is provided in the Supplementary Data (Fig. S3).

### Sample preparation for SEM

SEM of MTB cells was carried out with some modifications in the established protocol^63^. Microscopy was performed with a Zeiss Supra 55VP microscope (Carl Zeiss, Oberkochen, Germany). Secondary electron microscopic images were taken at low electron energy at 15 kV.

### Acid-Fast staining

The Auramine O stain was used in combination with Nile Red (9-diethylamino-5H-benzo[α]phenoxazine-5 -one) to detect the acid-fast property of cells by following an earlier established protocol^6^.

### Extraction of RNA from MTB for microarray analysis

RNA isolation from nitrite-treated and nontreated bacilli was performed at different time points using the TRIzol method^64^. Genes with >2 fold change and $p$-value ≤ 0.05 (i.e., 95% data confidence interval) were considered to be significantly up- or downregulated.

### Microarray data processing

In order to study the gene expression profile of nitrite-treated MTB, we availed the services of Genotypic India, Pvt., Ltd. RNA was used as a starting material, and samples (nitrite-treated and nontreated) were processed independently in duplicate. Total RNA was isolated using Qiagen’s RNeasy Minikit (Cat. #74104). From the total RNA (1 μg) extracted, 50 ng/μL RNA was further processed for microarray analysis. Single color labeling was performed, and an 8 × 15k array chip was used. Briefly, the RNA isolated from the samples was converted into cDNA and then into c-RNA with a Cy3-labeled probe using Agilent Quick Amp labeling kit. The random hexamer method of labeling was performed, followed by T7 promoter based linear amplification to generate labeled c-RNA (one-color microarray-based gene expression analysis). 600 ng of c-RNA was used for one array. For hybridization, Agilent *in situ* hybridization kit (part number 5188–5242) was used. RNA quality was checked using a bioanalyzer, which is a chip-based capillary electrophoresis machine. Probes were spotted on glass slides (eight arrays on one glass slide; each array has 60k probes). After hybridization, the slides were scanned using an Agilent microarray scanner and raw data were extracted using the Agilent Feature Extraction Software. Raw data were then used in GeneSpring GX 12.0 software for normalization by the percentile shift normalization method. Data were filtered on the basis of fold change in nitrite-treated cells as compared to untreated cells. Genes with > 1.5 fold change and $p$-value ≤ 0.05 (i.e., 95% data confidence interval) were considered to be significantly up- or downregulated. Data obtained from the series of microarray experiments were analyzed using mainly three bioinformatics tools: Database for Annotation, Visualization and Integrated Discovery (DAVID), KEGG pathway and Cytoscape, and TB Database (http://www.tbdb.org/). Using DAVID and KEGG, a functional analysis of differentially expressed genes (GO analysis) along with the analysis of signaling pathways was performed. These differentially expressed genes were compared with different dormancy models of H37Rv strain.

### Real-Time PCR/qPCR Study

For qPCR, 1 µg of total RNA was reverse-transcribed to cDNA using a single-strand synthesis kit (Sigma) as per the manufacturer’s instructions. qPCR was carried out using a QuantiTect SYBR Green PCR kit as per the manufacturer’s instructions. Gene-specific primers were used as mentioned in Table S7, where their respective amplification conditions are also mentioned. The transcript levels between various RNA samples were normalized using *sigA*. The experiments were carried out in triplicate for each time point.

## Supplementary information

Supplementary file 1

## References

[1] Global Tuberculosis report, W.H.O. (WHO) (2016)

[2] Bloom BR, Murray CJL (1999). Tuberculosis: commentary on a reemergent killer. Science.

[3] Rustad TR, Sherrid AM, Minch KJ, Sherman DR (2009). Hypoxia: a window into *Mycobacterium tuberculosis* latency. Cellular Microbiology.

[4] Global Tuberculosis report, W.H.O. (WHO) (2017)

[5] Ayrapetyan M, Williams TC, Baxter R, Oliver JD (2015). Viable but Nonculturable and Persister Cells Coexist Stochastically and Are Induced by Human Serum. Infect Immun..

[6] Deb C (2009). A Novel *in Vitro* Multiple-Stress Dormancy Model for *Mycobacterium tuberculosis* generates a Lipid-Loaded, Drug-Tolerant, Dormant Pathogen. Plos One..

[7] Betts J, Lukey P, Robb L, McAdam R, Duncan K (2002). Evaluation of a nutrient starvation model of *Mycobacterium tuberculosis* persistence by gene and protein expression profiling. Mol Microbiol..

[8] Voskuil M (2004). *Mycobacterium tuberculosis* gene expression during environmental conditions associated with latency. Tuberculosis..

[9] Wayne L, Hayes L (1996). An *in vitro* model for sequential study of shift down of *Mycobacterium tuberculosis* through two stages of nonreplicating persistence. Infect Immun..

[10] Salina EG (2014). Potassium availability triggers *Mycobacterium tuberculosis* transition to, and resuscitation from, non-culturable (dormant) states. Open Biology..

[11] Shleeva M, Mukamolova G, Young M, Williams H, Kaprelyants A (2004). Formation of ‘non-culturable’ cells of *Mycobacterium smegmatis* in stationary phase in response to growth under suboptimal conditions and their Rpf-mediated resuscitation. Microbiology..

[12] Dhillon, J., Lowri, E. D., Mitchson, D. *Mycobacterium tuberculosis* from chronic murine infections that grow in liquid but not on solid medium. *BMC infect*.*dis*. **4**(51), 10.1186/1471-2334-4-51 (2004). 10.1186/1471-2334-4-51 PMC534102 15548322

[13] Shleeva MO (2002). Formation and resuscitation of ‘non-culturable’ cells of and *Mycobacterium tuberculosis* in prolonged stationary phase. Microbiology..

[14] Demidenok O, Kaprelyants A, Goncharenko A (2014). Toxin–antitoxin vapBC locus participates in formation of the dormant state in *Mycobacterium smegmatis*. FEMS Microbiol Lett..

[15] Yarbrough,J., Rake, J. & Eagon, R. Bacterial Inhibitory Effects of Nitrite: Inhibition of Active Transport, But Not of Group Translocation, and of Intracellular Enzymes. *Applied And Environmental Microbiology*. doi: 0099-2240/80/04-0831/04$02.00/0 (1980). 10.1128/aem.39.4.831-834.1980 PMC291428 6769392

[16] Salina E, Mollenkopf H, Kaufmann S, Kaprelyants AM (2009). Tuberculosis Gene Expression during Transition to the “Non-Culturable” State. Acta Naturae..

[17] Medlab E, Bernstein S, Steward D (1952). A bacteriologic study of resected tuberculous lesions. Am. Rev. Tuberc..

[18] Gomez J, McKinney J (2004). Tuberculosis persistence, latency, and drug tolerance. Tuberculosis..

[19] Haapanen J, Kass I, Gensini G, Middlebrook G (1959). Studies on the gaseous content of tuberculous cavities. Am Rev Respir Dis..

[20] Wayne L, Sohaskey C (2001). Nonreplicating persistence of *Mycobacterium tuberculosis*. Annu Rev Microbiol..

[21] Steinera E (2014). CysK2 from *Mycobacterium tuberculosis* Is an O-Phospho-L-Serine-Dependent S-Sulfocysteine Synthase. J Bacteriol..

[22] Schnappinger D (2003). Transcriptional adaptation of *Mycobacterium tuberculosis* within macrophages: insights into the phagosomal environment. J Exp Med..

[23] Rohde K, Abramovitch R, Russell D (2007). *Mycobacterium tuberculosis* Invasion of Macrophages: Linking Bacterial Gene Expression to Environmental Cues. Cell Host & Microbe..

[24] Voskuil, M. I. *et al*. The response of *Mycobacterium tuberculosis* to reactive oxygen and nitrogen species. *Front Microbiol*, 10.3389/fmicb.2011.00105 (2011). 10.3389/fmicb.2011.00105 PMC3119406 21734908

[25] Rustad Tige R., Harrell Maria I., Liao Reiling, Sherman David R. (2008). The Enduring Hypoxic Response of Mycobacterium tuberculosis. PLoS ONE.

[26] Voskuil M, Visconti K, Schoolnik G (2004). *Mycobacterium tuberculosis* gene expression during adaptation to stationary phase and low-oxygen dormancy. Tuberculosis..

[27] Fisher M, Plikaytis B, Shinnick T (2002). Microarray Analysis of the *Mycobacterium tuberculosis* Transcriptional Response to the Acidic Conditions Found in Phagosomes. J. Bacteriol..

[28] Bussel A, Zhang T, Carl F (2013). Nitrite produced by *Mycobacterium tuberculosis* in human macrophages in physiologic oxygen impacts bacterial ATP consumption and gene expression. Proc Natl Acad Sci..

[29] Rodriguez G, Voskuil M, Gold B, Schoolnik G, Smith I (2002). ideR, an Essential Gene in *Mycobacterium tuberculosis*: Role of IdeR in Iron-Dependent Gene Expression, Iron Metabolism, and Oxidative stress response. Infection and Immunity..

[30] Sherman D (2001). Regulation of the *Mycobacterium tuberculosis* hypoxic response gene encoding α-crystallin. Proc Natl Acad Sci..

[31] Springer B (2001). Silencing of oxidative stress response in *Mycobacterium tuberculosis*: expression patterns of ahpC inactivation. Infect Immun..

[32] Master S (2002). Oxidative stress response genes in *Mycobacterium tuberculosis*: role of ahpC in resistance to peroxynitrite and stage-specific survival in macrophages. Microbiology.

[33] Sikri K (2015). The pleiotropic transcriptional response of *Mycobacterium tuberculosis* to vitamin C is robust and overlaps with the bacterial response to multiple intracellular stresses. Microbiology.

[34] Pinto R, Tang Q, Britton W, Leyh T, Triccas J (2004). The *Mycobacterium tuberculosis* cysD and cysNC genes form a stress-induced operon that encodes a tri-functional sulfate-activating complex. Microbiology.

[35] Zhang M (2005). Expression and characterization of the carboxyl esterase Rv3487c from *Mycobacterium tuberculosis*. Protein Expr Purification..

[36] Zimhony Oren, Schwarz Alon, Raitses-Gurevich Maria, Peleg Yoav, Dym Orly, Albeck Shira, Burstein Yigal, Shakked Zippora (2015). AcpM, the Meromycolate Extension Acyl Carrier Protein of Mycobacterium tuberculosis, Is Activated by the 4′-Phosphopantetheinyl Transferase PptT, a Potential Target of the Multistep Mycolic Acid Biosynthesis. Biochemistry.

[37] Yamamoto K, Muniruzzaman S, Rajagopalan M, Madiraju M (2002). Modulation of *Mycobacterium tuberculosis* DnaA protein–adenine-nucleotide interactions by acidic phospholipids. Biochem J..

[38] Andries K (2005). A diarylquinoline drug active on the ATP synthesis of *Mycobacterium tuberculosis*. Science.

[39] McKinney J (2000). Persistence of *Mycobacterium tuberculosis* in macrophages and mice requires the glyoxylate shunt enzyme isocitrate lyas. Nature.

[40] Lorenz G, Fink M (2001). The glyoxylate cycle is required for fungal virulence. Nature.

[41] Kumar Ranjeet, Sanyal Suparna (2012). Mycobacterium tuberculosis: Dormancy, Persistence and Survival in the Light of Protein Synthesis. Understanding Tuberculosis - Deciphering the Secret Life of the Bacilli.

[42] Gyan S, Shiohira Y, Sato I, Takeuchi M, Sato T (2006). Regulatory Loop between Redox Sensing of the NADH/NAD Ratio by Rex (YdiH) and Oxidation of NADH by NADH Dehydrogenase Ndh in *Bacillus subtilis*. Journal of Bacteriology.

[43] Sohaskey C, Wayne L (2003). Role of narK2X and narGHJI in hypoxic upregulation of nitrate reduction by *Mycobacterium tuberculosis*. J Bacteriol..

[44] Malm S (2009). The roles of the nitrate reductase NarGHJI, the nitrite reductase NirBD and the response regulator GlnR in nitrate assimilation of *Mycobacterium tuberculosis*. Microbiology.

[45] Kumar R (2009). Glyoxylate shunt: Combating Mycobacterium at forefront. International Journal of Integrative Biology..

[46] Samten B, Wang X, Barnes P (2009). *Mycobacterium tuberculosis* ESX-1 system-secreted protein ESAT-6 but not CFP10 inhibits human T-cell immune responses. Tuberculosis.

[47] Tan T, Lee W, Alexander D, Grinstein S, Liu J (2006). The ESAT-6/CFP-10 secretion system of Mycobacterium marinum modulates phagosome maturation. Cell Microbiol..

[48] McEvoy Christopher R. E., Cloete Ruben, Müller Borna, Schürch Anita C., van Helden Paul D., Gagneux Sebastien, Warren Robin M., Gey van Pittius Nicolaas C. (2012). Comparative Analysis of Mycobacterium tuberculosis pe and ppe Genes Reveals High Sequence Variation and an Apparent Absence of Selective Constraints. PLoS ONE.

[49] Tiwari Bhavana Mishra, Kannan Nisha, Vemu Lakshmi, Raghunand Tirumalai R. (2012). The Mycobacterium tuberculosis PE Proteins Rv0285 and Rv1386 Modulate Innate Immunity and Mediate Bacillary Survival in Macrophages. PLoS ONE.

[50] Sampson Samantha L. (2011). Mycobacterial PE/PPE Proteins at the Host-Pathogen Interface. Clinical and Developmental Immunology.

[51] Albrethsen Jakob, Agner Jeppe, Piersma Sander R., Højrup Peter, Pham Thang V., Weldingh Karin, Jimenez Connie R., Andersen Peter, Rosenkrands Ida (2013). Proteomic Profiling ofMycobacterium tuberculosisIdentifies Nutrient-starvation-responsive Toxin–antitoxin Systems. Molecular & Cellular Proteomics.

[52] Fozo E (2010). Abundance of type I toxin-antitoxin systems in bacteria: Searches for new candidates and discovery of novel families. Nucleic Acid Res..

[53] Makarova Kira S, Wolf Yuri I, Koonin Eugene V (2009). Comprehensive comparative-genomic analysis of Type 2 toxin-antitoxin systems and related mobile stress response systems in prokaryotes. Biology Direct.

[54] Pandey D, Gerdes K (2005). Toxin-antitoxin loci are highly abundant in free-living but lost from host-associated prokaryotes. Nucleic Acid Res..

[55] Zhu L (2006). Characterization of mRNA interferases from *Mycobacterium tuberculosis*. Biol Chem..

[56] Wayne L, Hayse L (1998). Nitrate reduction as a marker for hypoxic shift down of *Mycobacterium tuberculosis*. Tuber Lung Dis..

[57] Khomenko A (1987). The variability of *Mycobacterium tuberculosis* in patients with cavitary pulmonary tuberculosis in the course of chemotherapy. Tubercle..

[58] Gandhi NR (2006). Extensively drug resistant tuberculosis as a cause of death in patients co-infected with tuberculosis and HIV in a rural area of South Africa. Lancet.

[59] Shi L (2010). Carbon flux rerouting during *Mycobacterium tuberculosis* growth arrest. Mol Microbiol..

[60] Hutter T, Dick B (1998). Increased alanine dehydrogenase activity during dormancy in *Mycobacterium smegmatis*. FEMS Microbiol Lett..

[61] Monje-casas F (2001). Expression analysis of the nrdHIEF Operon from *Escherichia coli* conditions that trigger the transcript level *in vivo*. The Journal of Biological Chemistry..

[62] Akif M, Khare G, Tyagi A, Mande S, Sardesai A (2008). Functional Studies of Multiple Thioredoxins from *Mycobacterium tuberculosis*. Journal of bacteriology..

[63] Takayama K, Wang L, Merkal R (1973). Scanning Electron Microscopy of the H37Ra Strain of *Mycobacterium tuberculosis* Exposed to Isoniazid. Am Soc Microbiol..

[64] Akhtar S, Sarkar S, Mishra A, Sarkar D (2011). A method to extract intact and pure RNA from mycobacteria. Analytical Biochemistry..

[65] Voskuil M (2003). Inhibition of respiration by nitric oxide induces a *Mycobacterium tuberculosis* dormancy program. J Exp Med..

[66] Taneja Neetu Kumra, Dhingra Sakshi, Mittal Aditya, Naresh Mohit, Tyagi Jaya Sivaswami (2010). Mycobacterium tuberculosis Transcriptional Adaptation, Growth Arrest and Dormancy Phenotype Development Is Triggered by Vitamin C. PLoS ONE.

