# Supplementary material for: Evidence of nitrite acting as a stable and robust inducer of non-cultivability in Mycobacterium tuberculosis with physiological relevance
Source: Sci Rep. 2019 Jun 25;9:9261. doi: 10.1038/s41598-019-45652-8 (PMC6593118; doi:10.1038/s41598-019-45652-8)

Supplementary File

Title: Evidence of nitrite acting as a stable and robust inducer of non-cultivability in *Mycobacterium tuberculosis* with physiological relevance

Authors: Suwarna P Gample[1, 2], Sonia Agrawal[1,2], Dhiman Sarkar[1, 2#]

Table S1: Genes up regulated in *M. tuberculosis* after 1 day of nitrite exposure

Ra_ID	Probe Name	Gene Name	GeoMean_1D	P-Value
MRA_RS00705	Rv3118	*sseC1*	1.87	0.000
MRA_RS00785	Rv1854c	*Ndh*	2.33	0.000
MRA_RS04310	Rv1197	*esxK*	1.76	0.002
MRA_RS04500	Rv2256c	*Rv2256c*	1.69	0.002
MRA_RS05725	MTB000020	*Rrl*	2.00	0.002
MRA_RS01020	Rv2617c	*Rv2617c*	3.54	0.000
MRA_RS01030	Rv2391	*nirA*	1.58	0.000
MRA_RS01135	Rv2107	*pe22*	1.88	0.000
MRA_RS01180	Rv3614c	*Rv3614c*	3.59	0.000
MRA_RS08205	Rv1980c	*mpt64*	1.58	0.005
MRA_RS01345	Rv0896	*gltA2*	1.52	0.000
MRA_RS01470	Rv3290c	*lat*	2.84	0.001
MRA_RS01530	Rv0244c	*fadE5*	1.94	0.001
MRA_RS01535	Rv1793	*esxN*	1.83	0.001
MRA_RS07910	Rv3416	*whiB3*	2.17	0.004
MRA_RS01665	Rv3871	*Rv3871*	2.56	0.001
MRA_RS02155	Rv2392	*cysH*	1.51	0.001

Ra_ID	Probe Name	Gene Name	GeoMean_1D	P-Value
MRA_RS02195	Rv3616c	*Rv3616c*	3.72	0.001
MRA_RS02435	Rv3619c	*esxI*	2.00	0.001
MRA_RS05630	Rv3914	*trxC*	1.66	0.002
MRA_RS05705	Rv0188	*Rv0188*	2.02	0.002
MRA_RS02460	Rv3053c	*nrdH*	2.19	0.001
MRA_RS02495	Rv1198	*esxL*	2.30	0.001
MRA_RS02545	Rv1435c	*Rv1435c*	2.08	0.001
MRA_RS02550	Rv1405c	*Rv1405c*	2.42	0.001
MRA_RS02575	Rv2108	*ppe36*	1.55	0.001
MRA_RS03320	Rv2558	*Rv2558*	1.55	0.001
MRA_RS04005	Rv3205c	*Rv3205c*	1.69	0.001
MRA_RS04010	Rv2204c	*Rv2204c*	1.56	0.001
MRA_RS04145	Rv2496c	*pdhB*	2.01	0.001
MRA_RS04180	Rv0129c	*fbpC*	2.59	0.001
MRA_RS04745	Rv2590	*fadD9*	2.84	0.002
MRA_RS04790	Rv3620c	*esxM*	1.74	0.002
MRA_RS04920	Rv1076	*lipU*	1.52	0.002
MRA_RS04925	Rv1925	*fadD31*	1.51	0.002
MRA_RS04935	Rv3251c	*rubA*	4.69	0.002
MRA_RS06140	Rv3249c	*Rv3249c*	4.02	0.002
MRA_RS06185	Rv3250c	*rub*	4.60	0.002
MRA_RS06360	Rv3045	*adhC*	2.10	0.003
MRA_RS07775	Rv3241c	*Rv3241c*	2.37	0.004
MRA_RS06370	Rv3054c	*Rv3054c*	3.03	0.003

Ra_ID	Probe Name	Gene Name	GeoMean_1D	P-Value
MRA_RS06375	Rv3289c	*Rv3289c*	2.54	0.003
MRA_RS07780	Rv1698	*Rv1698*	1.52	0.004
MRA_RS06495	Rv2620c	*Rv2620c*	1.59	0.003
MRA_RS06895	Rv0485	*Rv0485*	2.43	0.003
MRA_RS06930	Rv3248c	*sahH*	2.27	0.003
MRA_RS07150	Rv2347c	*esxJ*	1.79	0.003
MRA_RS08460	Rv0928	*pstS3*	2.04	0.005
MRA_RS07315	MRA_2676	-	1.88	0.003
MRA_RS07405	Rv0491	*regX3*	1.80	0.003
MRA_RS07435	Rv3615c	*Rv3615c*	3.59	0.003
MRA_RS07470	Rv1221	*sigE*	1.89	0.003
MRA_RS07500	Rv1697	*Rv1697*	1.77	0.003
MRA_RS07540	Rv0211	*pckA*	2.08	0.004
MRA_RS07790	Rv2393	*Rv2393*	2.03	0.004
MRA_RS07800	Rv2927c	*Rv2927c*	2.10	0.004
MRA_RS08665	Rv0467	*icl*	2.27	0.005
MRA_RS08970	Rv1544	*Rv1544*	1.67	0.005
MRA_RS08975	Rv3613c	*Rv3613c*	1.99	0.005
MRA_RS09685	Rv2619c	*Rv2619c*	1.89	0.006
MRA_RS09465	Rv2334	*cysK1*	1.57	0.005
MRA_RS09485	Rv0251c	*hsp*	1.50	0.005
MRA_RS09495	Rv0140	*Rv0140*	2.81	0.006
MRA_RS09515	Rv2780	*ald*	3.14	0.006
MRA_RS11690	Rv2428	*ahpC*	1.82	0.008

Ra_ID	Probe Name	Gene Name	GeoMean_1D	P-Value
MRA_RS11980	Rv2707	*Rv2707*	2.14	0.008
MRA_RS11465	Rv1072	*Rv1072*	1.55	0.008
MRA_RS09820	Rv3052c	*nrdI*	2.22	0.006
MRA_RS09950	Rv1195	*pe13*	1.87	0.006
MRA_RS10020	Rv2499c	*Rv2499c*	1.74	0.006
MRA_RS10040	Rv1894c	*Rv1894c*	2.03	0.006
MRA_RS10165	Rv1466	*Rv1466*	1.96	0.006
MRA_RS10175	Rv0815c	*cysA2*	1.56	0.006
MRA_RS11165	Rv2202c	*adoK*	2.12	0.007
MRA_RS11170	Rv3252c	*alkB*	3.43	0.007
MRA_RS11205	Rv3463	*Rv3463*	3.15	0.007
MRA_RS11585	Rv3810	*pirG*	1.89	0.008
MRA_RS12345	Rv2466c	*Rv2466c*	1.95	0.009
MRA_RS12935	Rv2710	*sigB*	1.94	0.010
MRA_RS12475	Rv3051c	*nrdE*	2.07	0.009
MRA_RS13135	Rv2621c	*Rv2621c*	2.23	0.010
MRA_RS12710	Rv0784	*Rv0784*	2.00	0.009
MRA_RS13315	Rv2115c	*Rv2115c*	2.00	0.011
MRA_RS14145	Rv1577c	*Rv1577c*	1.56	0.013
MRA_RS14195	Rv0464c	*Rv0464c*	1.66	0.013
MRA_RS13675	Rv1987	*Rv1987*	1.60	0.012
MRA_RS14400	Rv1461	*Rv1461*	2.99	0.014
MRA_RS16230	Rv1196	*ppe18*	1.55	0.018

Ra_ID	Probe Name	Gene Name	GeoMean_1D	P-Value
MRA_RS14420	Rv2662	*Rv2662*	1.54	0.015
MRA_RS16240	Rv2160c	*Rv2160c*	1.73	0.018
MRA_RS14435	Rv1880c	*cyp140*	1.99	0.015
MRA_RS04305	Rv0309	*Rv0309*	1.51	0.019
MRA_RS17060	Rv1463	*Rv1463*	2.49	0.020
MRA_RS17145	Rv1375	*Rv1375*	2.26	0.021
MRA_RS17305	Rv1464	*csd*	1.90	0.022
MRA_RS17345	Rv3086	*adhD*	1.57	0.022
MRA_RS17290	Rv1462	*Rv1462*	2.26	0.022
MRA_RS17295	Rv0146	*Rv0146*	1.57	0.022
MRA_RS18185	Rv1419	*Rv1419*	1.53	0.024
MRA_RS20205	Rv0287	*esxG*	1.84	0.048
MRA_RS19155	Rv0288	*esxH*	1.80	0.026
MRA_RS20505	MTB000026	*rnpB*	2.45	0.049
MRA_RS17485	Rv3206c	*moeB1*	1.61	0.023
MRA_RS19185	Rv0848	*cysK2*	1.51	0.029
	Rv3874	*esxB*	1.66	0.065
	Rv3161c	*Rv3161c*	1.56	0.068
	Rv3019c	*esxR*	1.53	0.086

Table S2: Genes down regulated in *M. tuberculosis* after 1 day of nitrite exposure

Ra_ID	Probe Name	Gene Name	GeoMean_1D	P-Value
MRA_RS19190	Rv3621c	*ppe65*	-1.59	0.001
MRA_RS01815	Rv0342	*iniA*	-1.69	0.035

MRA_RS09625	Rv1815	*Rv1815*	-1.90	0.037
MRA_RS15885	Rv2990c	*Rv2990c*	-1.50	0.006
MRA_RS01495	Rv0280	*ppe3*	-1.53	0.015
MRA_RS00930	Rv0171	*mce1C*	-1.56	0.025

Table S3: Genes up regulated in *M. tuberculosis* after 3 day of nitrite exposure

Ra_ID	Probe Name	Gene Name	GeoMean_1D	P-Value
MRA_RS20415	Rv3849	*Rv3849*	4.80	0.000
MRA_RS05945	Rv1116	*Rv1116*	1.52	0.001
MRA_RS10920	Rv2063	Rv2063	2.70	0.001
MRA_RS19640	Rv3705A	*Rv3705A*	2.33	0.002
MRA_RS14445	Rv2712c	*Rv2712c*	1.48	0.002
MRA_RS09350	Rv1761c	*Rv1761c*	4.41	0.005
MRA_RS09355	Rv1762c	*Rv1762c*	5.07	0.012
MRA_RS00615	Rv0112	*gca*	1.51	0.007
MRA_RS09345	Rv1760	*Rv1760*	1.59	0.007
MRA_RS14625	Rv2748c	*ftsK*	1.57	0.008
MRA_RS20305	Rv3828c	*Rv3828c*	1.57	0.008
MRA_RS16390	Rv2564	*glnQ*	1.99	0.008
MRA_RS19220	Rv3627c	*Rv3627c*	1.50	0.009
MRA_1990			1.74	0.010
MRA_RS00370	Rv0066c	*icd2*	2.74	0.010
MRA_RS06285	Rv1179c	*Rv1179c*	1.50	0.011
MRA_RS12660	Rv2382c	*mbtC*	1.91	0.013

Ra_ID	Probe Name	Gene Name	GeoMean_1D	P-Value
MRA_RS12450	Rv2342	*Rv2342*	1.80	0.019
MRA_0412			2.45	0.020
MRA_RS12620	Rv2374c	*hrcA*	1.55	0.016
MRA_RS12605	Rv2371	*pe-pgrs40*	2.11	0.016
MRA_RS09545	Rv0468	*fadB2*	2.29	0.017
MRA_RS08975	Rv3613c	*Rv3613c*	1.58	0.020
MRA_RS12145	Rv2290	*lppO*	1.63	0.020
MRA_RS16395	Rv3084	*lipR*	2.25	0.020
MRA_RS18310	Rv3448	*Rv3448*	2.25	0.021
MRA_RS13960	Rv2159c	*Rv2159c*	1.68	0.022
MRA_RS03360	Rv0636	*Rv0636*	1.58	0.022
MRA_RS07915	Rv1489A	*Rv1489A*	1.64	0.025
MRA_RS10155	Rv1921c	*lppF*	1.90	0.025
MRA_RS19515	Rv3683	*Rv3683*	1.93	0.025
MRA_RS16995	Rv3196A	*Rv3196A*	2.05	0.025
MRA_RS04265	Rv0807	*Rv0807*	1.61	0.037
MRA_RS15115	Rv2844	*Rv2844*	1.79	0.027
MRA_RS12830	Rv2411c	*Rv2411c*	1.55	0.027
MRA_RS12380	Rv2330c	*lppP*	1.63	0.028
MTB000010		*lysT*	4.21	0.028
MRA_RS07975	Rv1500	*Rv1500*	1.62	0.030
MRA_RS08015	Rv1507A	*Rv1507A*	1.69	0.038

Ra_ID	Probe Name	Gene Name	GeoMean_1D	P-Value
MRA_RS13950	MTB000019	*rrs*	5.86	0.031
MRA_RS11030	Rv2082	*Rv2082*	2.70	0.033
MRA_RS19645	Rv3706c	*Rv3706c*	1.88	0.033
MRA_RS03315	Rv0631c	*recC*	1.85	0.033
MRA_RS03940	Rv0747	*pe-pgrs10*	2.45	0.034
	Rv0192A		2.28	0.036
MRA_RS04570	Rv0862c	*Rv0862c*	2.24	0.040
MRA_RS07425	Rv1396c	pe-pgrs25	1.78	0.042
MRA_RS03615	Rv0685	*tuf*	1.96	0.043
MRA_RS07980	Rv1501	*Rv1501*	1.58	0.044
	MTB000028	*metV*	3.82	0.046
MRA_RS03145	Rv0600c	*Rv0600c*	2.21	0.046
MRA_RS11855	Rv2234	*ptpA*	1.84	0.051
MRA_RS04280	Rv0810c	*Rv0810c*	3.59	0.052
MRA_RS15880	Rv2989	*Rv2989*	1.50	0.052
MRA_RS04715	Rv0891c	*Rv0891c*	2.09	0.054
MRA_RS05725	MTB000020	*rrl*	4.08	0.048
MRA_RS09565	Rv1803c	*pe-pgrs32*	1.54	0.044
MRA_RS04840	Rv0914c	*Rv0914c*	2.46	0.045
MRA_RS13550	Rv2541	*Rv2541*	1.77	0.045
MRA_RS03425	Rv0648	*Rv0648*	2.49	0.057
MRA_RS20390	Rv3845	*Rv3845*	1.79	0.051

Ra_ID	Probe Name	Gene Name	GeoMean_1D	P-Value
MRA_RS16900	Rv3180c	*Rv3180c*	1.73	0.055
MRA_RS01490	Rv0279c	*pe-pgrsS4*	1.80	0.056
MRA_RS18080	Rv3403c	*Rv3403c*	2.30	0.052
MRA_RS18600	Rv3507	*pe-pgrs53*	2.17	0.051
MRA_RS15035	Rv2828A	*Rv2828A*	1.53	0.042
	Rv1053c		1.63	0.044
MRA_RS06305	Rv1184c	*Rv1184c*	2.02	0.045
MRA_RS12015	Rv2264c	*Rv2264c*	2.42	0.047
MRA_RS11045	Rv2085	*Rv2085*	1.52	0.038
MRA_RS20340	Rv3835	*Rv3835*	1.53	0.034
	MTB000025	*leuU*	2.74	0.029
MRA_RS20405	Rv2706c	*Rv2706c*	1.67	0.021
MRA_RS13830	Rv2595	*Rv2595*	1.84	0.031
MRA_RS12250	Rv2308	*Rv2308*	1.62	0.004
MRA_RS14955	Rv2813	*Rv2813*	2.36	0.012
MRA_RS10885	Rv2056c	*rpsN2*	1.52	0.012
MRA_RS19580	Rv3695	*Rv3695*	1.98	0.012
MRA_RS12075	Rv2276	*cyp121*	1.92	0.010
MRA_RS06535	Rv1230c	*Rv1230c*	2.00	0.014
MRA_RS05780	Rv1087	*pe-pgrs21*	1.52	0.014
MRA_RS12340	Rv1595	*nadB*	1.99	0.014
MRA_1587			1.59	0.015

Ra_ID	Probe Name	Gene Name	GeoMean_1D	P-Value
MRA_RS05670	Rv1065	*Rv1065*	1.69	0.017
	Rv3344c	*pe-pgrs49*	1.62	0.017
	MTB000043	*metU*	1.76	0.017
	MTB000003	*leuT*	1.99	0.017
MRA_RS03985	Rv0755c	*ppe12*	1.72	0.018
MRA_RS13045	Rv2450c	*rpfE*	2.05	0.018
MRA_RS14075	Rv2641	*cadI*	4.94	0.020
MRA_RS12345	Rv2466c	*Rv2466c*	1.97	0.021
MRA_RS04505	Rv0849	*Rv0849*	3.08	0.023
MRA_RS20505	MTB000026	*rnpB*	3.33	0.024
MRA_RS04495	Rv0847	*lpqS*	3.14	0.027
MRA_RS15735	Rv2963	*Rv2963*	2.57	0.027
MRA_RS13135	Rv2621c	*Rv2621c*	2.52	0.028
MRA_RS14080	Rv2642	*Rv2642*	3.04	0.030
MRA_RS19185	Rv0848	*cysK2*	3.74	0.032

Table S4: Genes down regulated in *M. tuberculosis* after 3 day of nitrite exposure

Ra_ID	Probe Name	Gene Name	GeoMean_1D	P-Value
MRA_RS00060	Rv0009	*ppiA*	-1.75	0.010
MRA_RS19190	Rv3621c	*ppe65*	-2.04	0.062
MRA_RS03735	Rv0707	*rpsC*	-1.81	0.015

Ra_ID	Probe Name	Gene Name	GeoMean_1D	P-Value
MRA_RS07360	Rv1384	*carB*	-1.76	0.014
MRA_RS03720	Rv0704	*rplB*	-1.70	0.002
MRA_RS02435	Rv3619c	*esxI*	-1.72	0.038
MRA_RS02495	Rv1198	*esxL*	-1.83	0.007
MRA_RS02715	Rv0516c	*Rv0516c*	-1.53	0.044
MRA_RS03575	Rv0677c	*mmpS5*	-1.91	0.007
MRA_RS03655	Rv0692	*Rv0692*	-1.99	0.081
MRA_RS03700	Rv0700	*rpsJ*	-2.27	0.062
MRA_RS09970	Rv1884c	*rpfC*	-1.61	0.055
MRA_RS01305	Rv0243	*fadA2*	-1.81	0.085
MRA_RS04355	Rv0824c	*desA1*	-1.79	0.012
MRA_RS03710	Rv0702	*rplD*	-1.83	0.011
MRA_RS03705	Rv0701	*rplC*	-1.78	0.019
MRA_RS03810	Rv0722	*rpmD*	-1.60	0.002
MRA_RS00930	Rv0171	*mce1C*	-1.77	0.036
MRA_RS05355	Rv1010	*ksgA*	-1.57	0.015
MRA_RS18830	Rv3549c	*Rv3549c*	-1.60	0.014
MRA_RS19330	Rv3648c	*cspA*	-1.97	0.002
MRA_RS05835	Rv1094	*desA2*	-1.82	0.035
MRA_RS07235	Rv1360	*Rv1360*	-1.91	0.029
MRA_RS07315	MRA_2676	-	-1.76	0.026
MRA_RS16105	Rv3029c	*fixA*	-2.10	0.002

Ra_ID	Probe Name	Gene Name	GeoMean_1D	P-Value
MRA_RS18280	Rv3442c	*rpsI*	-1.55	0.053
MRA_RS18285	Rv3443c	*rplM*	-1.57	0.036
MRA_RS20370	Rv3841	*bfrB*	-2.51	0.056
MRA_RS20780	Rv3922c	*Rv3922c*	-1.60	0.025
MRA_RS20785	Rv3923c	rnpA	-1.98	0.035
MRA_RS09900	Rv1870c	*Rv1870c*	-1.57	0.006
MRA_RS09950	Rv1195	*pe13*	-1.61	0.015
MRA_RS09195	Rv1738	*Rv1738*	-3.43	0.046
MRA_RS03795	Rv0719	*rplF*	-1.99	0.008
MRA_RS07885	Rv1484	*inhA*	-1.88	0.003
MRA_RS10195	Rv1929c	*Rv1929c*	-1.62	0.015
MRA_RS09625	Rv1815	*Rv1815*	-1.99	0.045
MRA_RS10755	Rv2031c	*hspX*	-1.78	0.006
MRA_RS10760	Rv2032	*Acg*	-2.04	0.036
MRA_RS13910	Rv2610c	*pimA*	-1.53	0.027
MRA_RS13995	Rv2626c	*Rv2626c*	-1.68	0.024
MRA_RS14005	Rv2628	*Rv2628*	-2.53	0.033
MRA_RS12865	Rv2418c	*Rv2418c*	-1.51	0.039
MRA_RS17445	Rv3280	*accD5*	-1.53	0.035
MRA_RS17810	Rv3351c	*Rv3351c*	-1.63	0.042
MRA_RS00920	Rv0169	*mce1A*	-1.99	0.025
MRA_RS16230	Rv1196	*ppe18*	-1.97	0.037

Ra_ID	Probe Name	Gene Name	GeoMean_1D	P-Value
MRA_RS16620	Rv3127	*Rv3127*	-1.90	0.008
MRA_RS16640	Rv3130c	*Rv3130c*	-1.89	0.051
MRA_RS16645	Rv3131	*Rv3131*	-2.19	0.011

Table S5: Genes up regulated in *M. tuberculosis* after 6 day of nitrite exposure

Ra_ID	Probe Name	Gene Name	GeoMean_1D	P-Value
MRA_RS01005	Rv0186	*bglS*	1.77	0.002
MRA_RS01020	Rv2617c	*Rv2617c*	3.15	0.001
MRA_RS04145	Rv2496c	*pdhB*	1.60	0.003
MRA_RS01470	Rv3290c	*lat*	2.04	0.007
MRA_RS05705	Rv0188	*Rv0188*	1.93	0.004
MRA_RS01720	Rv0323c	*Rv0323c*	1.68	0.012
MRA_RS10315	Rv1952	*Rv1952*	1.82	0.029
MRA_RS05125	Rv0968	*Rv0968*	1.53	0.004
MRA_RS09485	Rv0251c	*hsp*	2.97	0.003
MRA_RS06500	Rv1223	*htrA*	2.12	0.001
MRA_RS09515	Rv2780	*ald*	1.77	0.010
MRA_RS06370	Rv3054c	*Rv3054c*	3.10	0.003
MRA_RS06375	Rv3289c	*Rv3289c*	1.82	0.011
MRA_RS10095	Rv1909c	*furA*	1.52	0.048
MRA_RS10550	Rv1992c	*ctpG*	2.83	0.008
MRA_RS10555	Rv1993c	*Rv1993c*	1.54	0.010

Ra_ID	Probe Name	Gene Name	GeoMean_1D	P-Value
MRA_RS14085	Rv2643	*arsC*	2.18	0.004
MRA_RS14400	Rv1461	*Rv1461*	1.84	0.001
MRA_RS17305	Rv1464	*csd*	1.66	0.003
MRA_RS17495	Rv1222	*Rv1222*	2.03	0.006
MRA_RS12935	Rv2710	*sigB*	2.94	0.023
MRA_RS05725	MTB000020	*rrl*	1.59	0.058
MRA_RS17060	Rv1463	*Rv1463*	1.74	0.000
MRA_RS17155	Rv3223c	*sigh*	1.60	0.029
MRA_RS17250	Rv1955	*Rv1955*	1.63	0.001
MRA_RS20505	MTB000026	*rnpB*	1.72	0.005
MRA_RS19580	Rv3695	*Rv3695*	1.75	0.040
MRA_RS14075	Rv2641	*cadI*	3.33	0.003
MRA_RS19185	Rv0848	*cysK2*	2.09	0.001

Table S6: Genes down regulated in *M. tuberculosis* after 6 day of nitrite exposure

Ra_ID	Probe Name	Gene Name	GeoMean_1D	P-Value
MRA_RS11920	Rv2245	*kasA*	-2.73	0.000
MRA_RS11370	Rv2144c	*Rv2144c*	-1.69	0.000
MRA_RS01815	Rv0342	*iniA*	-2.04	0.000
MRA_RS03520	Rv0667	*rpoB*	-2.08	0.000
MRA_RS02435	Rv3619c	*esxI*	-1.99	0.000
MRA_RS07885	Rv1484	*inhA*	-1.87	0.000

**Ra_ID**	**Probe Name**	**Gene Name**	**GeoMean_1D**	**P-Value**
MRA_RS16105	Rv3029c	*fixA*	-2.15	0.000
MRA_RS20150	Rv3799c	*accD4*	-1.57	0.000
MRA_RS11915	Rv2244	*acpM*	-2.67	0.000
MRA_RS11925	Rv2246	*kasB*	-2.23	0.000
MRA_RS00060	Rv0009	*ppiA*	-1.83	0.000
MRA_RS04685	Rv0885	*Rv0885*	-1.81	0.000
MRA_RS08695	Rv1641	*infC*	-2.01	0.001
MRA_RS19190	Rv3621c	*ppe65*	-1.69	0.001
MRA_RS03700	Rv0700	*rpsJ*	-2.48	0.002
MRA_RS02495	Rv1198	*esxL*	-2.04	0.001
MRA_RS09690	Rv1828	*Rv1828*	-1.51	0.001
MRA_RS01810	Rv0341	*iniB*	-3.75	0.001
MRA_RS01535	Rv1793	*esxN*	-1.96	0.001
MRA_RS18105	Rv3408	*Rv3408*	-1.97	0.001
MRA_RS04790	Rv3620c	*esxM*	-2.17	0.001
MRA_RS02450	Rv1827	*cfp17*	-1.59	0.001
MRA_RS07150	Rv2347c	*esxJ*	-2.28	0.001
MRA_RS20785	Rv3923c	*rnpA*	-2.13	0.001
MRA_RS11175	Rv2109c	*prcA*	-1.86	0.001
MRA_RS11785	Rv2222c	*glnA2*	-1.54	0.001
MRA_RS07405	Rv0491	*regX3*	-1.59	0.001
MRA_RS09970	Rv1884c	*rpfC*	-1.62	0.001

Ra_ID	Probe Name	Gene Name	GeoMean_1D	P-Value
MRA_RS16620	Rv3127	*Rv3127*	-1.95	0.001
MRA_RS03605	Rv0683	*rpsG*	-1.77	0.002
MRA_RS09520	Rv1794	*Rv1794*	-2.13	0.002
MRA_RS13005	Rv2442c	*rplU*	-1.98	0.002
MRA_RS13080	Rv2457c	*clpX*	-2.38	0.002
MRA_RS15865	Rv2986c	*hupB*	-1.79	0.002
MRA_RS06930	Rv3248c	*sahH*	-1.61	0.002
MRA_RS00930	Rv0171	*mce1C*	-1.94	0.002
MRA_RS09950	Rv1195	*pe13*	-1.86	0.002
MRA_RS02535	Rv0483	*lprQ*	-2.25	0.002
MRA_RS07235	Rv1360	*Rv1360*	-2.39	0.002
MRA_RS17445	Rv3280	*accD5*	-2.08	0.002
	MTB000008	*trpT*	-1.90	0.002
MRA_RS18155	Rv3418c	*groES*	-1.56	0.002
MRA_RS11910	Rv2243	*fabD*	-2.07	0.002
MRA_RS03670	Rv0695	*Rv0695*	-1.79	0.003
MRA_RS04310	Rv1197	*esxK*	-2.05	0.003
MRA_RS16215	Rv3049c	*Rv3049c*	-1.53	0.003
MRA_RS16645	Rv3131	*Rv3131*	-1.76	0.003
MRA_RS03795	Rv0719	*rplF*	-2.13	0.003
MRA_RS09910	Rv1872c	*lldD2*	-2.28	0.004
MRA_RS00920	Rv0169	*mce1A*	-1.96	0.002

Ra_ID	Probe Name	Gene Name	GeoMean_1D	P-Value
MRA_RS09625	Rv1815	*Rv1815*	-2.86	0.003
MRA_RS03735	Rv0707	*rpsC*	-1.68	0.003
MRA_RS08460	Rv0928	*pstS3*	-1.52	0.003
MRA_RS12160	Rv2293c	*Rv2293c*	-1.53	0.004
MRA_RS15125	Rv2846c	*efpA*	-1.58	0.004
MRA_RS20780	Rv3922c	*Rv3922c*	-2.04	0.004
MRA_RS19160	Rv1594	*nadA*	-1.86	0.004
MRA_RS14005	Rv2628	*Rv2628*	-2.08	0.004
MRA_RS03720	Rv0704	*rplB*	-1.80	0.005
MRA_RS19865	Rv3749c	*Rv3749c*	-2.10	0.005
MRA_RS03385	Rv0640	*rplK*	-1.64	0.005
MRA_RS11930	Rv2247	*accD6*	-1.82	0.005
MRA_RS09965	Rv1883c	*Rv1883c*	-1.70	0.005
MRA_RS02305	Rv0440	*groEL2*	-1.70	0.005
MRA_RS15095	Rv2840c	*Rv2840c*	-2.73	0.005
MRA_RS17055	Rv0932c	*pstS2*	-1.84	0.005
MRA_RS04355	Rv0824c	*desA1*	-2.19	0.005
MRA_RS10470	Rv1981c	*nrdF1*	-1.67	0.004

Ra_ID	Probe Name	Gene Name	GeoMean_1D	P-Value
MRA_RS03710	Rv0702	*rplD*	-2.15	0.006
MRA_RS18370	Rv3460c	*rpsM*	-1.97	0.006
MRA_RS19330	Rv3648c	*cspA*	-2.34	0.006
MRA_RS17390	Rv3269	*Rv3269*	-1.77	0.006
MRA_RS05835	Rv1094	*desA2*	-2.01	0.006
MRA_RS09195	Rv1738	*Rv1738*	-2.73	0.006
MRA_RS15430	Rv2904c	*rplS*	-1.61	0.004
MRA_RS03705	Rv0701	*rplC*	-1.88	0.006
MRA_RS03715	Rv0703	*rplW*	-1.53	0.006
MRA_RS03615	Rv0685	*Tuf*	-1.71	0.006
MRA_RS03610	Rv0684	*fusA1*	-1.51	0.006
	Rv3874	*esxB*	-1.66	0.008
MRA_RS07035	Rv1322A	*Rv1322A*	-1.78	0.009
MRA_RS18365	Rv3459c	*rpsK*	-1.86	0.008
MRA_RS20790	Rv3924c	*rpmH*	-1.51	0.007
MRA_RS16230	Rv1196	*ppe18*	-1.77	0.007
MRA_RS09905	Rv1871c	*Rv1871c*	-2.05	0.007
MRA_RS19195	Rv3622c	*pe32*	-1.59	0.004
MRA_RS13320	Rv3281	*Rv3281*	-1.69	0.007
MRA_RS12480	Rv2348c	*Rv2348c*	-1.76	0.007
MRA_RS08700	Rv1642	*rpmI*	-1.73	0.010
MRA_RS11485	Rv2166c	*Rv2166c*	-1.57	0.010

Ra_ID	Probe Name	Gene Name	GeoMean_1D	P-Value
MRA_RS03730	Rv0706	*rplV*	-1.59	0.012
MRA_RS10755	Rv2031c	*hspX*	-1.80	0.013
MRA_RS06275	Rv1177	*fdxC*	-1.65	0.013
MRA_RS11375	Rv2145c	*wag31*	-1.64	0.013
MRA_RS09900	Rv1870c	*Rv1870c*	-1.64	0.011
MRA_RS00005	Rv0001	*dnaA*	-1.59	0.016
MRA_RS06260	Rv1174c	*TB8.4*	-1.51	0.014
MRA_RS16640	Rv3130c	*Rv3130c*	-1.62	0.014
MRA_RS19870	Rv3750c	*Rv3750c*	-1.63	0.019
	MTB000021	*rrf*	-1.55	0.017
MRA_RS20400	Rv3846	*soda*	-1.52	0.017
MRA_RS03575	Rv0677c	*mmpS5*	-1.91	0.018
MRA_RS16060	Rv3020c	*esxS*	-1.52	0.020
MRA_RS20545	Rv3875	*esxA*	-1.74	0.021
MRA_RS08705	Rv1643	*rplT*	-1.70	0.021
MRA_RS05745	Rv1080c	*greA*	-2.43	0.031
MRA_RS18380	Rv3462c	*infA*	-1.80	0.031
MRA_RS20370	Rv3841	*bfrB*	-2.44	0.026
MRA_RS03655	Rv0692	*Rv0692*	-2.01	0.027
MRA_RS20205	Rv0287	*esxG*	-1.92	0.028

Table S7: Details of the primers used in Real Time PCR study

Gene	Primer Sequence [a]	Amplicon Size (bp)	Annealing Temp[b](°C)
sigA	F: AAACCATCTGCTGGAAGCCA<br>R: CGGCCGGTGTAGCGC	160	55
hspX	F: ATCCCGGCCCACCTTCGACA<br>R: AGCACCTACCGGCAGCGACA	196	55
rv0031	F: AAGCCGATATCCAAGCCTGG<br>R: TTCCGAGTTGATGGGTGGC	108	55
rv0633	F: TGTTTATGGAGCCCCACGTC<br>R: AGCCGAAGTTCCACGACATC	166	55
ctpG	F: GAGCACGCGAACACTATCAG<br>R: TCAGCAACACTCCGGAGAAT	164	55
iniA	F: CAGGAATTCACCGAACCCGA<br>R: CAGGAATTCACCGAACCCGA	187	60
rv2621	F: GGAGACGATCGACCACTTGG<br>R: CCCGCATGATCCCTAAGTGG	167	60
cysK2	F: ACCCTGACCGTGGATTTTGG<br>R: GTACACCTTACCAGCGAGGG	182	60
rubA	F:GGTCTGCGACTACGTCTACGAC<br>R: TCGAAATCGACCTTCTCGCG	132	60
kas A	F: GGTCACCTCAAGGATCCGGT<br>R: GCTCTCGACAATCCTCTCGG	192	60

[a]Primers were designed using the IDT (Integrated DNA Technology) primer designer software.

[b]Initial denaturation of 95°C for 4 minutes followed by 40 cycles of 94°C (30 seconds), primer specific annealing temp. (30 seconds), 72°C (30 seconds) and final extension of 10 minutes at 72°C were used for amplification of mentioned genes followed by melting curve analysis.

Figure S1: Venn diagram representing number of genes overlapping at different time points in nitrite treated *M. tuberculosis* cells. Overlapping genes **A)** down regulated and **B)** up regulated in nitrite treated *M. tuberculosis* cells at day1, 3 and 6 respectively.

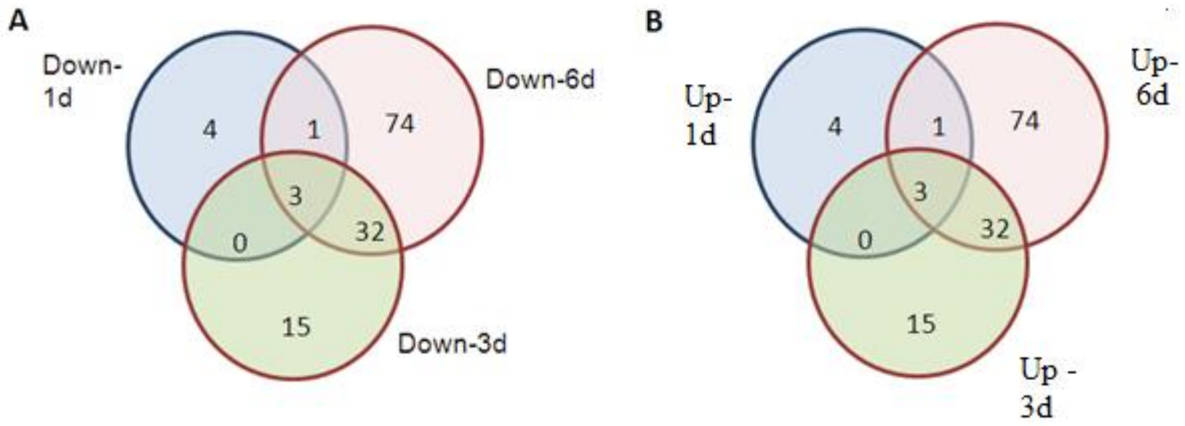

Figure S2: Standardization of live- dead analysis assay.

The cells were stained with SYTO9/ PI and observed under fluorescence microscope at 60X objective (EVOS, Invitrogen). It was observed that, log phase cells fluoresces green due to SYTO 9 staining (100% live) (Figure S1- A). While isopropanol treated cells, fluoresces yellow due to both SYTO 9 and PI staining (100% killed) (Figure S1- B). The scale bar corresponds to 50µm. Further, for the percent viability detection, a standard curve was drawn by spiking log phase cells with isopropanol treated cells in different ratio (1:0, 1:3, 1:1, 3:1 and 0:1) (Figure S1- C). The percent viability of nitrite treated and untreated cells were determined at different time intervals by using standard curve.

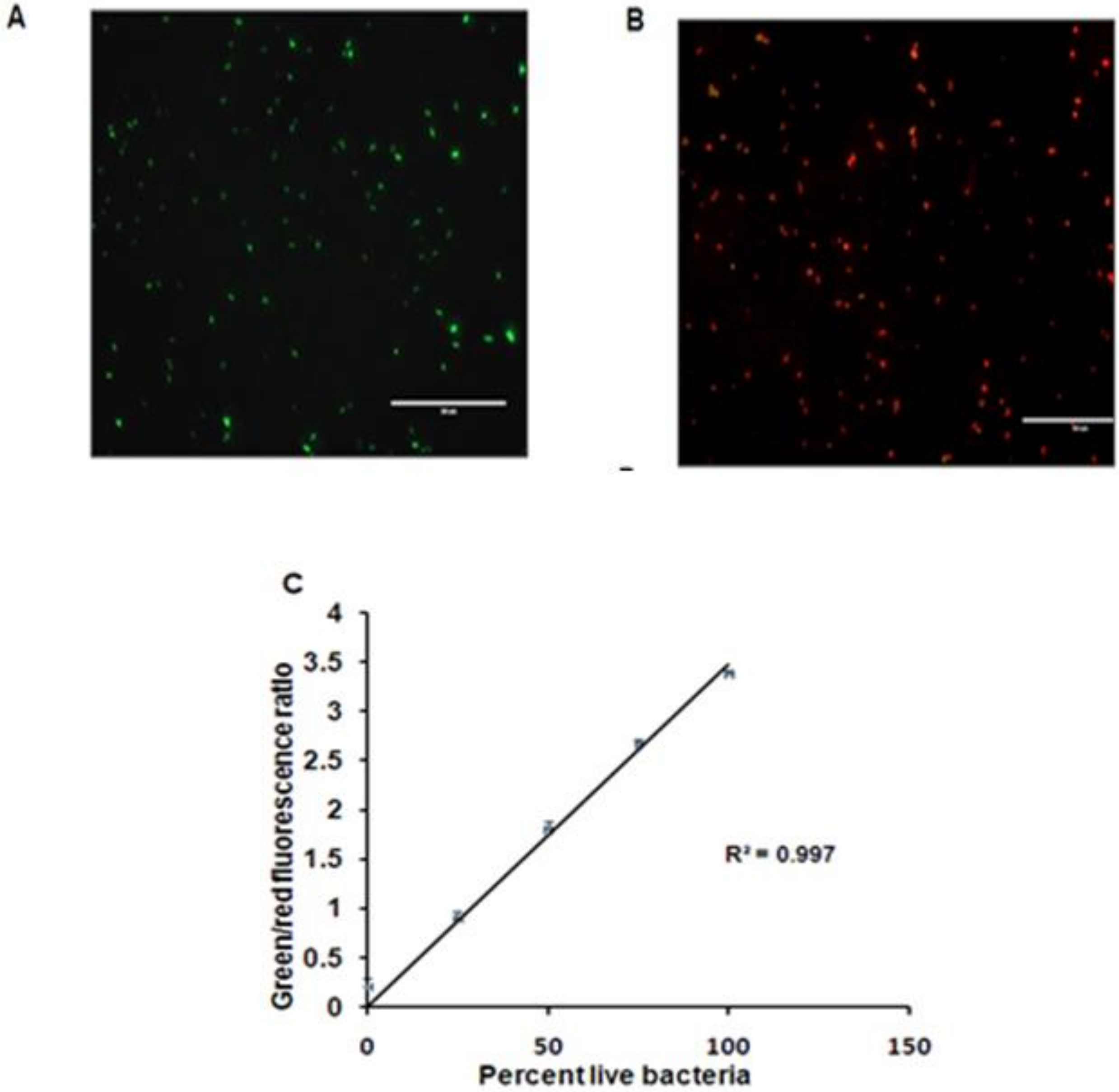

Figure S3: Live-dead analysis for isoniazid treatment. Log-phase cells were grown in *M.phlei* medium up to the optical density 0.2 (620 nm) and treated with Isoniazid (10µg/ml). The samples (200µl) were incubated with SYTO 9 (5.1µM) and PI (80µM) for 15mins. The fluorescence images were taken on different day like 0day, 1day, 3day, 6day and 10day by using the EVOS microscope (Life technology, Germany) (Figure S3).

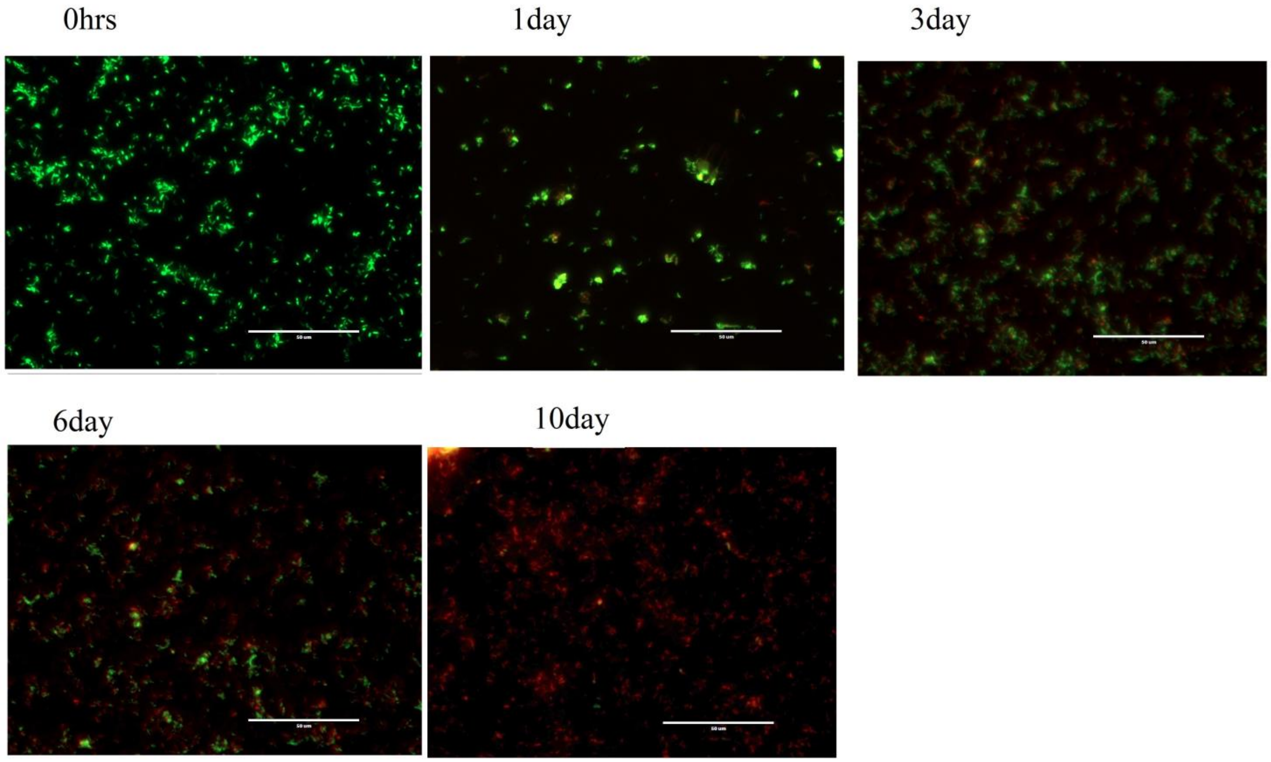

Supplement: Supplementary file 1 [file 41598_2019_45652_MOESM1_ESM.pdf]
